# Nonmonotonic recruitment of ventromedial prefrontal cortex during remote memory recall

**DOI:** 10.1371/journal.pbio.2005479

**Published:** 2018-07-02

**Authors:** Daniel N. Barry, Martin J. Chadwick, Eleanor A. Maguire

**Affiliations:** 1 Wellcome Centre for Human Neuroimaging, Institute of Neurology, University College London, London, United Kingdom; 2 Institute of Behavioural Neuroscience, Department of Experimental Psychology, Division of Psychology and Language Sciences, University College London, London, United Kingdom; University of Birmingham, United Kingdom of Great Britain and Northern Ireland

## Abstract

Systems-level consolidation refers to the time-dependent reorganisation of memory traces in the neocortex, a process in which the ventromedial prefrontal cortex (vmPFC) has been implicated. Capturing the precise temporal evolution of this crucial process in humans has long proved elusive. Here, we used multivariate methods and a longitudinal functional magnetic resonance imaging (fMRI) design to detect, with high granularity, the extent to which autobiographical memories of different ages were represented in vmPFC and how this changed over time. We observed an unexpected time course of vmPFC recruitment during retrieval, rising and falling around an initial peak of 8–12 months, before reengaging for older 2- and 5-year-old memories. This pattern was replicated in 2 independent sets of memories. Moreover, it was further replicated in a follow-up study 8 months later with the same participants and memories, for which the individual memory representations had undergone their hypothesised strengthening or weakening over time. We conclude that the temporal engagement of vmPFC in memory retrieval seems to be nonmonotonic, revealing a complex relationship between systems-level consolidation and prefrontal cortex recruitment that is unaccounted for by current theories.

## Author summary

Our past experiences are captured in autobiographical memories that allow us to recollect events from our lives long after they originally occurred. A part of the brain’s frontal lobe, called the ventromedial prefrontal cortex (vmPFC), is known to be important for supporting autobiographical memories, especially as memories become more remote. The precise temporal profile of the vmPFC’s involvement is unclear, yet this information is vital if we are to understand how memories change over time and the mechanisms involved. In this study, we sought to establish the time course of vmPFC engagement in the recollection of autobiographical memories while participants recalled memories of different ages during functional magnetic resonance imaging (fMRI). Using a method that detects the brain activity patterns associated with individual memories, we found that memory-specific neural patterns in vmPFC became more distinct over the first few months after a memory was formed, but then this initial involvement of vmPFC subsided after 1 year. However, more remote memories (2 years and older) appeared to reengage vmPFC once again. This temporal profile is difficult to accommodate within any single existing theory. Consequently, our results provoke a rethink about how memories evolve over time and the role played by the vmPFC.

## Introduction

We possess a remarkable ability to retrieve, with ease, one single experience from a lifetime of memories. How these individual autobiographical memories are represented in the brain over time is a central question of memory neuroscience that remains unanswered.

Consolidation takes place on two levels, which differ on both a spatial and temporal scale. On a cellular level, the stabilisation of new memory traces through modification of synaptic connectivity takes only a few hours [1] and is heavily dependent upon the hippocampus [2–5]. On a much longer timescale, the neocortex integrates new memories, a form of consolidation termed ‘systems-level’ [6]. The precise timeframe of this process is unknown. A related long-standing debate that has contributed to this uncertainty is whether or not the hippocampus ever relinquishes its role in autobiographical memory retrieval. One theory asserts that the hippocampus is not involved in the retrieval of memories after they have become fully consolidated to the neocortex [7]. Alternate views maintain that vivid, detailed autobiographical memories retain a permanent reliance on the hippocampus for their expression [8–12].

An undisputed feature of systems-level consolidation, however, is the strengthening of neural representations in the neocortex over time. Clarity on the time course of systems-level consolidation is therefore more likely to be achieved through scrutiny of its neocortical targets. While theoretical accounts often fail to specify these cortical locations, animal experiments have consistently implicated the medial prefrontal cortex. While this region has been associated with the formation [13, 14] and recall of recently acquired memories [15–17], in rodents, it appears to be disproportionately involved in the retrieval of memories learned weeks previously [18–26]. The dependency on this region, which emerges over time, is facilitated by postlearning activation [27] and structural changes [28–30].

The evolutionary expansion of prefrontal cortex in humans makes it challenging to make direct anatomical comparisons with rodents, but the ventromedial prefrontal cortex (vmPFC) has been proposed as a homologous site of long-term memory consolidation [31]. It may appear surprising that an association between impaired autobiographical memory retrieval and vmPFC lesions has only recently started to be more precisely characterised [32]. However, there are a number of confounding factors in this field [33]—nonselectivity of vmPFC lesions, methodological differences in memory elicitation, and the tendency of patients with vmPFC damage to recollect events that have never occurred, a phenomenon known as confabulation [34].

Numerous functional magnetic resonance imaging (fMRI) studies of vmPFC activity during autobiographical memory recall have been conducted but with inconclusive results. Delay-dependent increases in retrieval-related activity have been observed in some studies [35, 36] but not others [37–39]. Autobiographical memory, in particular, induces robust vmPFC engagement [40], but it is unclear whether this activity increases [41], decreases [42], or remains constant in accordance with memory remoteness [43–52].

A powerful method of fMRI analysis that can help to bridge the empirical gap between the human and animal literatures is multivoxel pattern analysis (MVPA), because of its increased sensitivity to specific neural representations [53]. Using this approach, Bonnici and colleagues [54] demonstrated that remote 10-year-old autobiographical memories were more detectable in the vmPFC than recent 2-week-old autobiographical memories, consistent with its proposed role as a long-term consolidation site. This difference was not apparent in other cortical areas, nor did it emerge from a standard univariate analysis. A follow-up study 2 years later with the same participants and memories demonstrated that the original 2-week-old memories were now as detectable in the vmPFC as the remote memories [55]. This suggested the recent memories had been fully consolidated in the vmPFC after just 2 years and perhaps even sooner.

The identification of this 2-year time window represented an opportunity to resolve the time course of systems-level consolidation with high precision. To do so, we sampled memories from 4-month intervals spanning a 2-year period and compared their neural representations using fMRI. As opposed to the pattern-classification approach employed by Bonnici and colleagues [54] to decode the neural signatures of individual memories, we used representational similarity analysis (RSA) [56]. This method compares the consistency of neural patterns across repetitions of a single memory against all other unrelated memories to detect its unique informational content in a region of interest (ROI). Differences in the strength of memory representations across time periods were interpreted as delay-dependent engagement of the vmPFC. To verify observed time-sensitive differences, we followed the neural evolution of individual memories in a follow-up study with the same participants and memories 8 months later. The selection of numerous time points characterised the consolidation process with unprecedented temporal resolution, while the longitudinal design was an opportunity not only to replicate these findings but to observe systems-level consolidation in action.

Systems-level consolidation is generally assumed to be an incremental process; therefore, we considered a gradual linear trajectory of vmPFC recruitment as the most likely outcome. The alternative hypothesis was a rapid strengthening of vmPFC neural representations in the first few months after an event. The results conformed to neither scenario and revealed an unexpected temporal relationship—a transient recruitment of the vmPFC beginning in the months following the initial experience, followed by an enduring signature of more remote memories. The second, longitudinal experiment confirmed this finding. This is the first demonstration, to our knowledge, of such a temporal dissociation in vmPFC-mediated memory retrieval.

## Results

### Experiment 1

**Fig 1:**
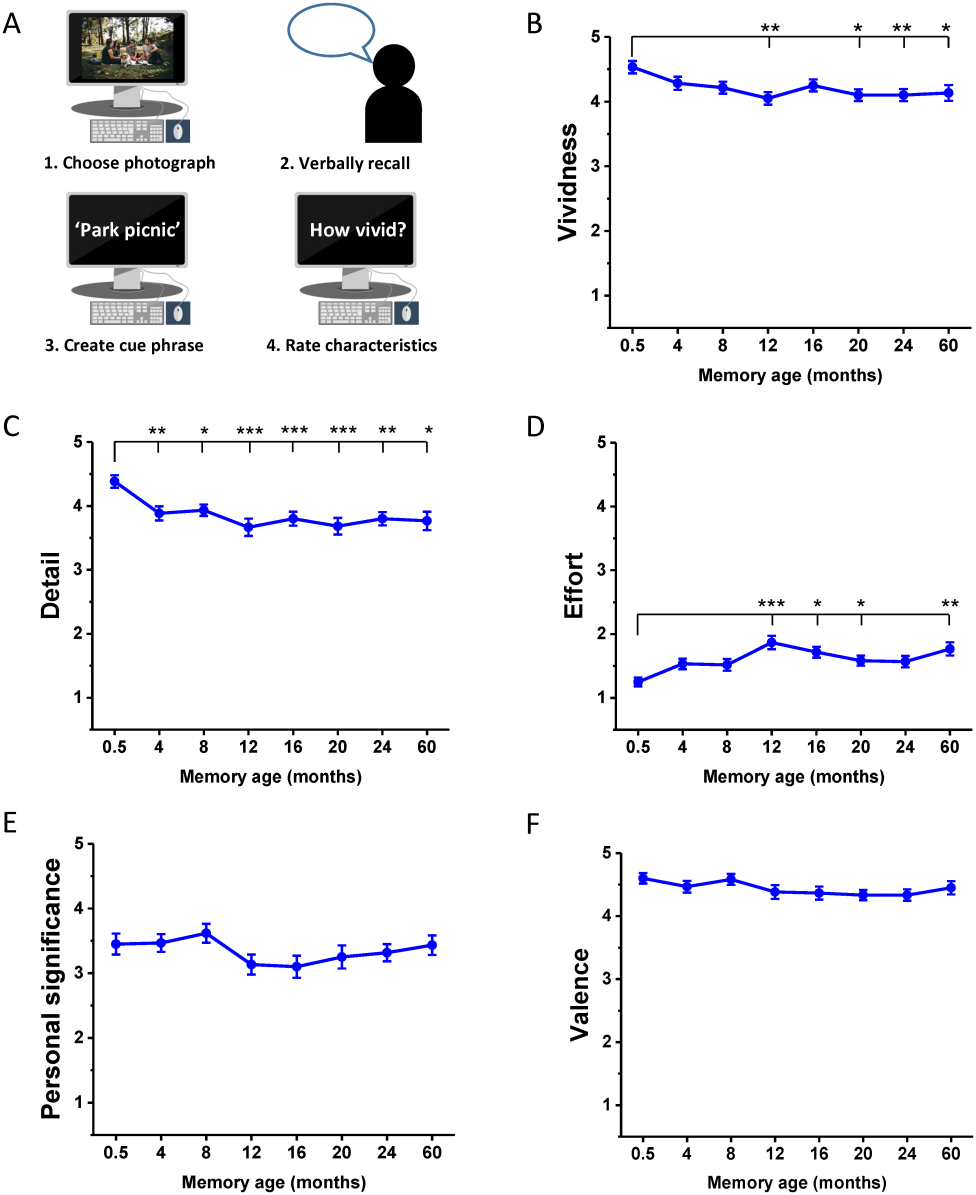
Memory harvesting and subjective ratings. (A) Schematic of the interview in which the autobiographical memories were harvested. Participants recalled a memory—which was cued by a personal photograph—chose a phrase to help remind them of this memory during the subsequent scanner task, and rated its characteristics. (B-F) Subjective ratings (means +/− 1 SEM; see also means and SDs in Table A in S1 Table, and S1 Data for individual ratings across both sets of memories) of memory characteristics at each time period for Experiment 1, averaged across the two sets of memories. Ratings were on a scale of 1 to 5, in which 1 was low and 5 was high. For emotional valence: 1–2 = negative, 3 = neutral, 4–5 = positive. * *p* < 0.05, ** *p* < 0.01, *** *p* < 0.001.

One week prior to the fMRI scan, with the assistance of personal photographs, participants (*n* = 30) verbally recalled and rated the characteristics of autobiographical memories from 8 time periods: memories that were 0.5 months old (0.5 M, i.e., 2-week-old memories), 4 M, 8 M, 12 M, 16 M, 20 M, 24 M, and also 60 M old—these latter memories serving as a definitive benchmark for remote (5-year-old) memories (see Materials and methods, Fig 1A). Two memories from each time period that were sufficiently vivid, detailed, specific, and unique in time and place were chosen for subsequent recall in the scanner. This meant that there were 2 full sets of memories. Participants created a short phrase pertaining to each autobiographical memory, which was paired with the photograph to facilitate recall during the subsequent fMRI scan.

#### Comparable subjective recall phenomenology across memories

While all memories satisfied the criteria of being vivid and detailed, and the ratings were high (Fig 1; see means and SDs in Table A in S1 Table), subjective vividness nevertheless varied as a function of memory age (F_(7,203)_ = 3.45, *p* = 0.002), with the most recent (0.5 M old) memories rated higher than 12 M (t_29_ = 4.08, *p* = 0.009), 20 M (t_29_ = 3.88, *p* = 0.016), 24 M (t_29_ = 4.18, *p* = 0.007), and 60 M old memories (t_29_ = 3.45, *p* = 0.049, Fig 1B). Subjective ratings of detail also differed across time points (F_(7,203)_ = 5.74, *p* < 0.001); once again, the most recent 0.5 M old memories were rated higher than 4 M (t_29_ = 4.45, *p* = 0.003), 8 M (t_29_ = 3.97, *p* = 0.012), 12 M (t_29_ = 5.00, *p* < 0.001), 16 M (t_29_ = 4.96, *p* < 0.001), 20 M (t_29_ = 5.37, *p* < 0.001), 24 M (t_29_ = 4.51, *p* = 0.003), and 60 M old memories (t_29_ = 3.98, *p* = 0.012, Fig 1C). The expenditure of effort during recall also varied according to remoteness of memories (F_(7,203)_ = 5.79, *p* < 0.001), with 0.5 M old memories being easier to recollect than 12 M (t_29_ = −5.29, *p* < 0.001), 16 M (t_29_ = −3.90, *p* = 0.015), 20 M (t_29_ = −3.67, *p* = 0.027), and 60 M old memories (t_29_ = −4.55, *p* = 0.003, Fig 1D). No significant difference was observed across time periods from 4 M to 60 M on any of these characteristics (all *p* > 0.05), nor did memories differ in their personal significance (F_(7,203)_ = 1.66, *p* = 0.120, Fig 1E) or emotional valence (F_(7,203)_ = 1.51, *p* = 0.166, Fig 1F) as a function of age.

In addition to these main ratings of interest, no difference was reported in the extent to which memories were recalled as active or static (F_(7,203)_ = 1.36, *p* = 0.224) or from a first- or third-person perspective (F_(3.69,107.02)_ = 1.09, *p* = 0.365) across time periods. The reported frequency at which memories were recalled since the original event (rated on a 5-point scale from ‘never’ to ‘very frequently’) differed as a function of time (F_(5.11,148.04)_ = 4.36, *p* < 0.001), with the most recent 0.5 M old memories thought about more frequently than 12 M (t_29_ = 4.37 *p* = 0.004), 16 M (t_29_ = 3.47, *p* = 0.046), and 24 M (t_29_ = 3.71, *p* = 0.024) old memories (see S11 Data for individual ratings for these characteristics).

Overall, therefore, memories were generally well matched on subjective phenomenological ratings and satisfied the criteria of high quality of memory recall, with only small differences observed for the most recent 0.5 M old memories compared to the other autobiographical memories, as might be expected.

#### Consistent level of details recalled across memories

**Fig 2:**
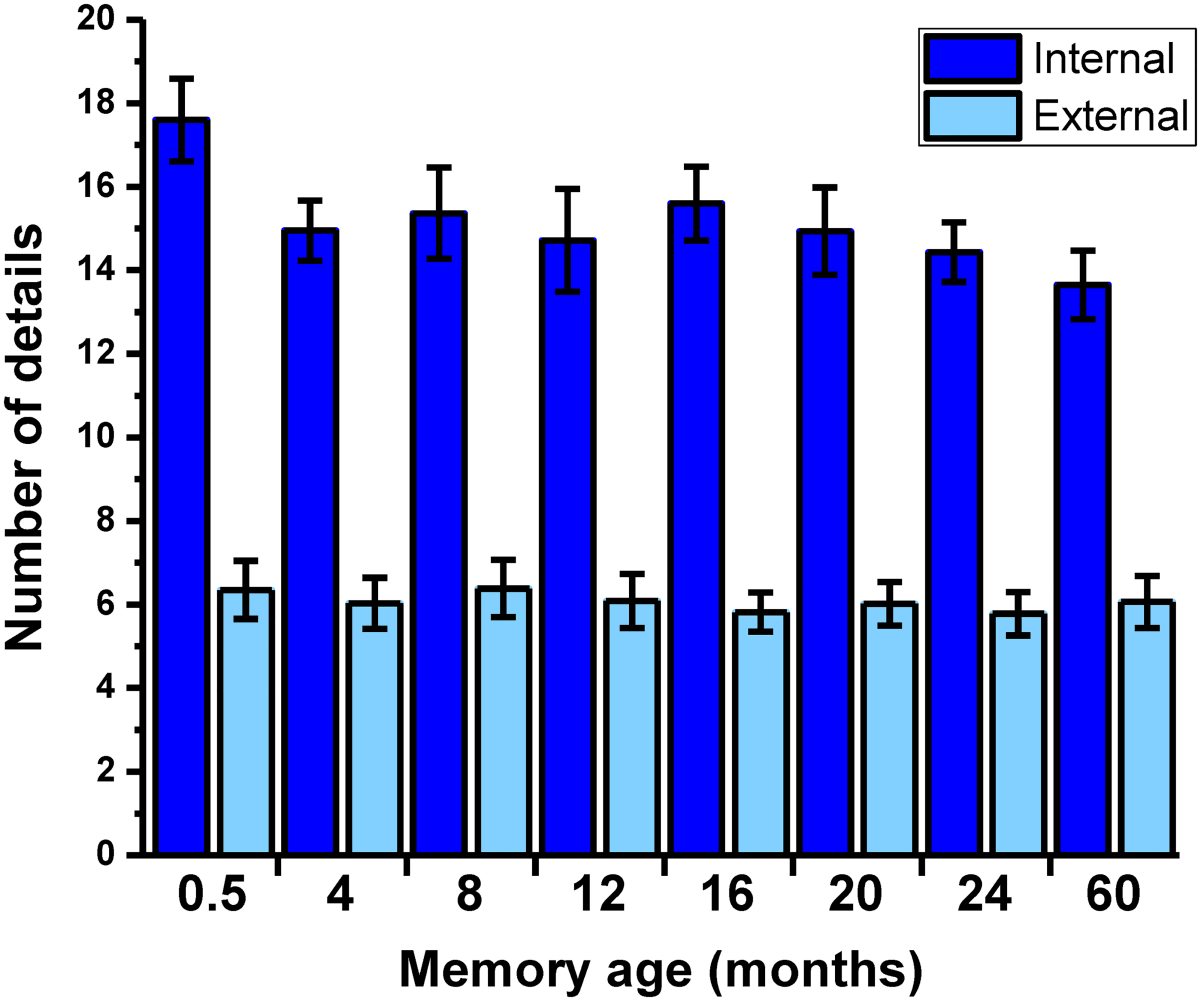
Objective scores for memory details. The mean +/− 1 SEM (see also means and SDs in Table B in S1 Table, and S2 Data for individual participant scores) number of internal and external details at each time period, averaged across the two sets of autobiographical memories.

To complement the subjective ratings of memory characteristics with a more objective assessment of their content, transcripts of participants’ memory interviews were scored using the autobiographical interview protocol ([57]; Materials and methods). In total for this first experiment, 10,187 details were scored. The mean (SD) number of internal details (bound to the specific ‘episodic’ spatiotemporal context of the event) and external details (arising from a general ‘semantic’ knowledge or references to unrelated events) are shown in Table B in S1 Table (see also Fig 2). They were then compared across time periods. In contrast to the subjective ratings of memory detail, the number of details recalled across memories from different time periods displayed only a nonsignificant trend (F_(4.54,131.66)_ = 1.92, *p* = 0.101). As expected, the number of internal and external details differed (F_(1,29)_ = 206.03, *p* < 0.001), with more internal details recalled for every time period (all *p* < 0.001). No interaction between time period and type of detail was observed (F_(7,203)_ = 1.87, *p* = 0.077). While a more targeted contrast of the most recent (0.5 M) and most remote (60 M) memories did reveal that 0.5 M events contained more internal details (t_29_ = 3.40, *p* = 0.002), this is consistent with participants’ subjective ratings and implies that any observed strengthening of neural representations over time could not be attributable to greater detail at remote time points. The number of external details recalled was remarkably consistent across all time periods, emphasising the episodic nature of recalled events irrespective of remoteness. Interrater reliabilities for the scoring (see Materials and methods) were high for both internal (intraclass correlation [ICC] = 0.94) and external (ICC = 0.81) details.

#### vmPFC engagement during recall was nonmonotonic

**Fig 3:**
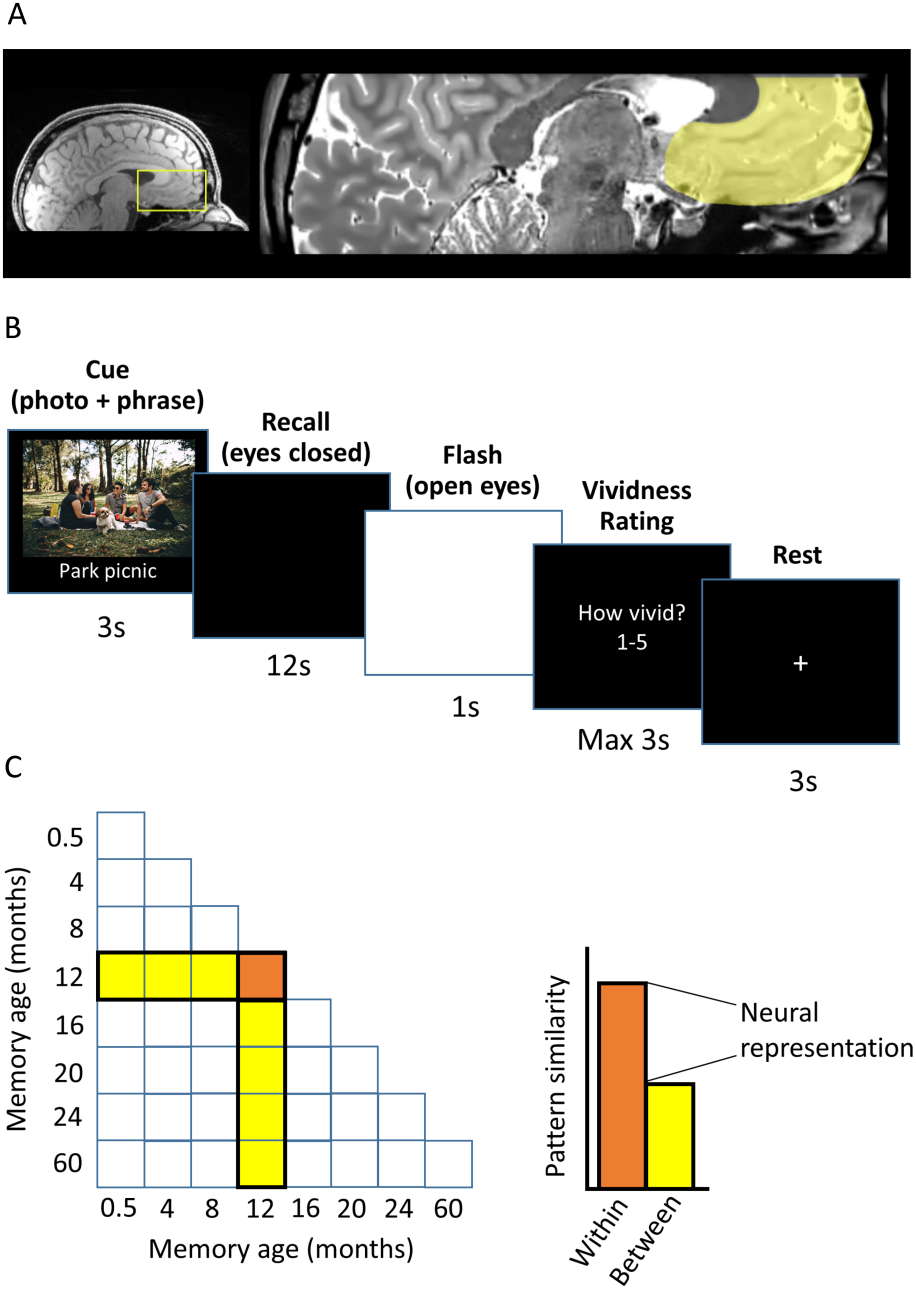
Experimental details. (A) The vmPFC is highlighted on an example participant’s structural MRI scan. (B) The timeline of an example trial from the scanning task. (C) Graphical illustration of the neural representation score calculation using RSA. The neural pattern similarity across trials recalling the same memory (orange) minus the mean pattern similarity between that memory and other memories (yellow) generates a ‘neural representation’ score. A score significantly higher than 0 indicates a neural pattern distinct to that memory is present in the vmPFC. RSA, representational similarity analysis; vmPFC, ventromedial prefrontal cortex.

vmPFC was delineated as the ventromedial surface of the frontal lobe and the medial portion of the orbital frontal cortex [58]. This comprises areas implicated in memory consolidation [31, 54, 55], namely Brodmann areas (BAs) 14, 25, ventral parts of 24 and 32, the caudal part of 10, and the medial part of BA 11 (Fig 3A, and Materials and methods).

On each trial, the photograph and associated preselected cue phrase relating to each event were displayed on a screen for 3 seconds. Following removal of this cue, participants then closed their eyes and recalled the memory. After 12 seconds, the black screen flashed white twice to cue the participant to open their eyes. The participant was then asked to rate how vivid the memory recall had been, using a 5-key button box, on a scale of 1–5, in which 1 was not vivid at all, and 5 was highly vivid (Fig 3B).

We used RSA to quantify the extent to which the strength of memory representations in the vmPFC differed as a function of memory age. This was achieved by contrasting the similarity of neural patterns when recalling the same memory with their similarity to other memories to yield a ‘neural representation’ score for each memory (see Materials and methods, Fig 3C). As there were 2 memories recalled per time period, the neural representation scores were averaged to produce 1 value for that time period.

**Fig 4:**
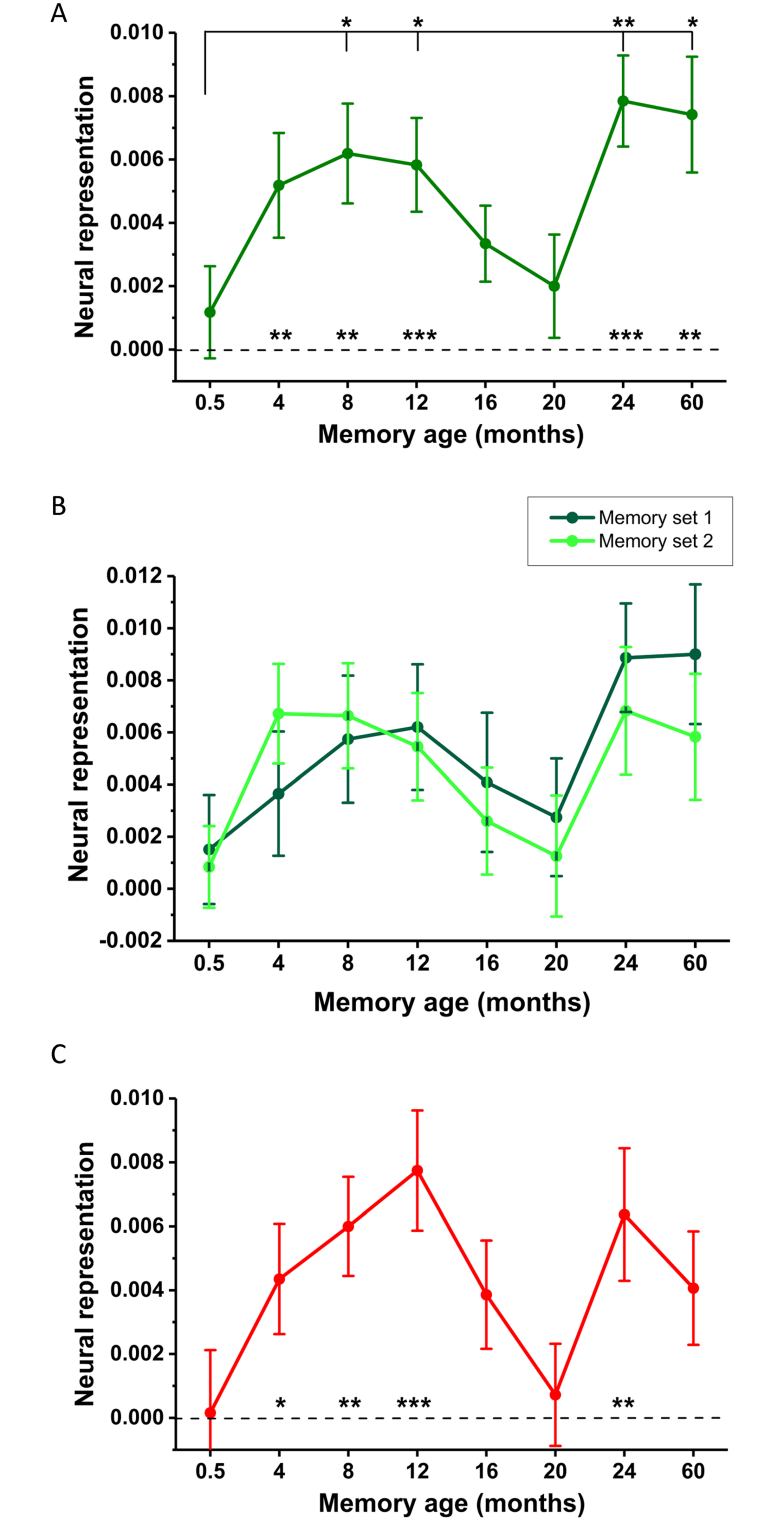
fMRI results of Experiment 1. (A) Mean +/− 1 SEM neural representation scores at each time point averaged across the two sets of memories. Asterisks above the dotted line indicate detectability of memories in vmPFC at each time point. Asterisks above the solid line indicate significant increases in memory representations compared to the most recent (0.5 M old) memories. * *p* < 0.05, ** *p* < 0.01, *** *p* < 0.001. See S1 Fig for the underlying RSM and S2 Fig for a box plot distribution of these data. (B) Neural representation scores at each time point plotted separately for the two sets of autobiographical memories. (C) Neural representation scores when using a single identically aged memory as a baseline. See S3 Data for individual participant scores. fMRI, functional magnetic resonance imaging; RSM, representational similarity matrix; vmPFC, ventromedial prefrontal cortex.

We anticipated an increase in the strength of memory representations at some point between 0.5 M and 24 M, in line with the results of Bonnici and Maguire [55]. This is what we observed, in which the most recent 0.5 M memories were undetectable (t_29_ = 0.72, *p* = 0.477) in vmPFC, in contrast to the distinct neural signatures observed for 4 M (t_29_ = 2.85, *p* = 0.008), 8 M (t_29_ = 3.09, *p* = 0.004), and 12 M (t_29_ = 3.66, *p* < 0.001) old memories (Fig 4A). These changes in the strength of memory representations were significant across time periods (F_(7,203)_ = 2.22, *p* = 0.034), with an observed increase in vmPFC recruitment from 0.5 M to 8 M (t_29_ = 2.07, *p* = 0.048) and 12 M (t_29_ = −2.20, *p* = 0.036).

However, what was observed for the following two time periods was unexpected—an apparent disengagement of the vmPFC over the next 8 months as we observed weak detectability of memory representations in vmPFC for 16 M (t_29_ = 1.85, *p* = 0.074) and 20 M (t_29_ = 1.03, *p* = 0.310) old memories. Neither 16 M (t_29_ = −1.06, *p* = 0.298) nor 20 M memories (t_29_ = −0.40, *p* = 0.691) were more strongly represented than the recent 0.5 M old memories. In contrast, the more remote 24 M (t_29_ = 4.34, *p* < 0.001) and 60 M (t_29_ = 3.55, *p* = 0.001) memories were detectable in the vmPFC and significantly more so than the most recent memories (24 M versus 0.5 M, t_29_ = −2.93, *p* = 0.007; 60 M versus 0.5 M, t_29_ = −2.54, *p* = 0.017) as well as the more temporally proximal 20 M old memories (24 M versus 20 M, t_29_ = −2.50, *p* = 0.018; 60 M versus 20 M, t_29_ = −2.32, *p* = 0.028).

The experimental design afforded us the opportunity to verify this nonmonotonic pattern. As we sampled 2 memories per time point, this time-dependent pattern should be evident in both sets of memories. As shown in Fig 4B, the two sets of memories followed a similar time course of changes in representation within vmPFC. This is a compelling replication, given that the two memories from each time period were unrelated in content as a prerequisite for selection, recalled in separate sessions in the scanner, and analysed independently from each other.

The availability of 2 memories at each time point also permitted the use of an alternative approach to calculating neural representation scores. Instead of using the similarity to memories from other time points as a baseline, we could also assess if memories were similar to their temporally matched counterpart in the other set. As can be seen in Fig 4C, the nonmonotonic pattern is preserved even when just using 1 identically aged memory as a baseline. In other words, the distinguishable patterns are specific to each individual memory rather than attributable to general retrieval processes associated with any memory of the same age.

An alternative explanation for memory representation scores that decreased over time is that the neural patterns became increasingly similar to memories from other time points rather than less consistent across repetitions, perhaps again reflecting more general retrieval processes. However, as evident in S3 Fig, between-memory scores remained stable across all time points and did not differ in their statistical significance (F_(5.24,152.02)_ = 1.72, *p* = 0.13). If anything, there was a slight trend for higher between-memory scores to accompany higher within-memory scores. Therefore, the detectability of neural representations appeared to be driven by consistent within-memory neural patterns.

#### The observed temporal relationship is unique to vmPFC

Our main focus was the vmPFC, given previous work highlighting specifically this region’s role in representing autobiographical memories over time [54, 55]. We also scanned within a partial volume (to attain high spatial resolution with a reasonable volume repetition time [TR]) and so were constrained in what other brain areas were available for testing (see Materials and methods). Nevertheless, we examined the same brain areas as Bonnici and colleagues [54] and Bonnici and Maguire [55], additionally including the precuneus, given its role in autobiographical memory retrieval [59], and in no case did we observe a significant change in memory detectability across time periods—entorhinal/perirhinal cortex (F_(7,203)_ = 1.55, *p* = 0.154), hippocampus (F_(7,203)_ = 0.98, *p* = 0.445), posterior parahippocampal cortex (F_(7,203)_ = 1.41, *p* = 0.202), retrosplenial cortex (F_(7,203)_ = 0.69, *p* = 0.682), temporal pole (F_(7,203)_ = 1.78, *p* = 0.093), lateral temporal cortex (F_(4.86,141.03)_ = 0.68, *p* = 0.636), or precuneus (F_(7,203)_ = 0.789, *p* = 0.562). Of note, memories that were undetectable in the vmPFC were still represented in other brain regions at these time points (see S2 Table for neural representation score means and SDs and S13 Data for individual participant scores). For example, 20-month-old memories that did not appear to recruit the vmPFC during retrieval were represented in the majority of other regions comprising the core autobiographical memory network (precuneus, lateral temporal cortex, parahippocampal cortex, and approaching significance in the retrosplenial cortex [t_29_ = 1.83, *p* = 0.08]).

Following scanning, participants completed 3 additional ratings. They were asked to indicate the extent to which the memories were changed by the 6 repetitions during scanning on a scale ranging from 1 (not at all) to 5 (completely). They reported that the memories were not changed very much by repetition (mean: 2.61, SD: 0.74). They were also asked how often during scanning they thought about the memory interview 1 week previous on a scale of 1 (not at all) to 5 (completely), with participants indicating they rarely thought about the interview (mean: 2.29, SD: 1.01). Finally, participants were asked the extent to which the recall of memories from each time period unfolded in a consistent manner over the course of the session. A difference was observed (F_(7,203)_ = 2.78, *p* = 0.009), with the most recent 0.5 M old memories being rated as more consistently recalled than the most remote 60 M memories (t_29_ = 3.97, *p* = 0.012).

In addition to the ROI-based approach, a searchlight analysis was also conducted in Montreal Neurological Institute (MNI) group normalised space to localise areas within the vmPFC where memories displayed high detectability across participants (see Materials and methods). We discovered a significant bilateral cluster of 652 voxels (see Fig A in S4 Fig) and subsequently used RSA to quantify the strength of neural representations at each time point within this area (see Fig B in S4 Fig). The results were highly similar to the whole-ROI analysis in native space, suggesting the main result may be driven by more spatially confined activity within the vmPFC. However, a searchlight approach is suboptimal to answer the current research question, as it requires an a priori model representational similarity matrix (RSM) against which to compare the neural patterns at each searchlight sphere, whereas the ROI approach makes no such assumptions.

We also conducted a standard mass-univariate analysis on the whole volume with memory remoteness as a parametric regressor, and no area displayed either a significant increase or decrease in activity in accordance with memory age, consistent with the findings of Bonnici and colleagues [54]. In a similar parametric analysis, we did not find evidence of the modulation of univariate activity by in-scanner vividness ratings, as might be suggested by the findings of Sheldon and Levine [60]; however, all memories chosen for the current study were highly vivid in nature.

One concern when studying covert cognitive processes such as autobiographical memory in the fMRI scanner is participant compliance, because performance is subjectively reported rather than objectively assessed. However, if participants were complying with task demands, there should be an association between in-scanner subjective ratings and the detectability of neural representations. When nonvivid trials were additionally incorporated into the RSA analysis, the mean memory representation score in the vmPFC for all participants averaged across time points decreased from 0.0049 (SD 0.005) to 0.0044 (SD 0.005). In fact, the deleterious effect of including these extra nonvivid trials was evident in 24 out of the 30 participants. Such a consistent relationship between participants’ subjective ratings of their own memory performance and the sensitivity of the RSA analysis to detect memory representations strongly suggests participants were performing the task as instructed.

### Rationale and predictions for Experiment 2

The nonmonotonic pattern we observed in the fMRI data did not manifest itself in the subjective or objective behavioural data. In fact, the only difference in those data was higher ratings for the most recent 0.5 M old memories. However, these were paradoxically the most weakly represented memories in the vmPFC, meaning the neural patterns were not driven by memory quality. The objective scoring of the memories confirmed comparable levels of detail provided for all memories, without any significant drop in episodic detail or increase in the amount of semantic information provided as a function of time. Therefore, the amount or nature of the memory details were not contributing factors.

**Fig 5:**
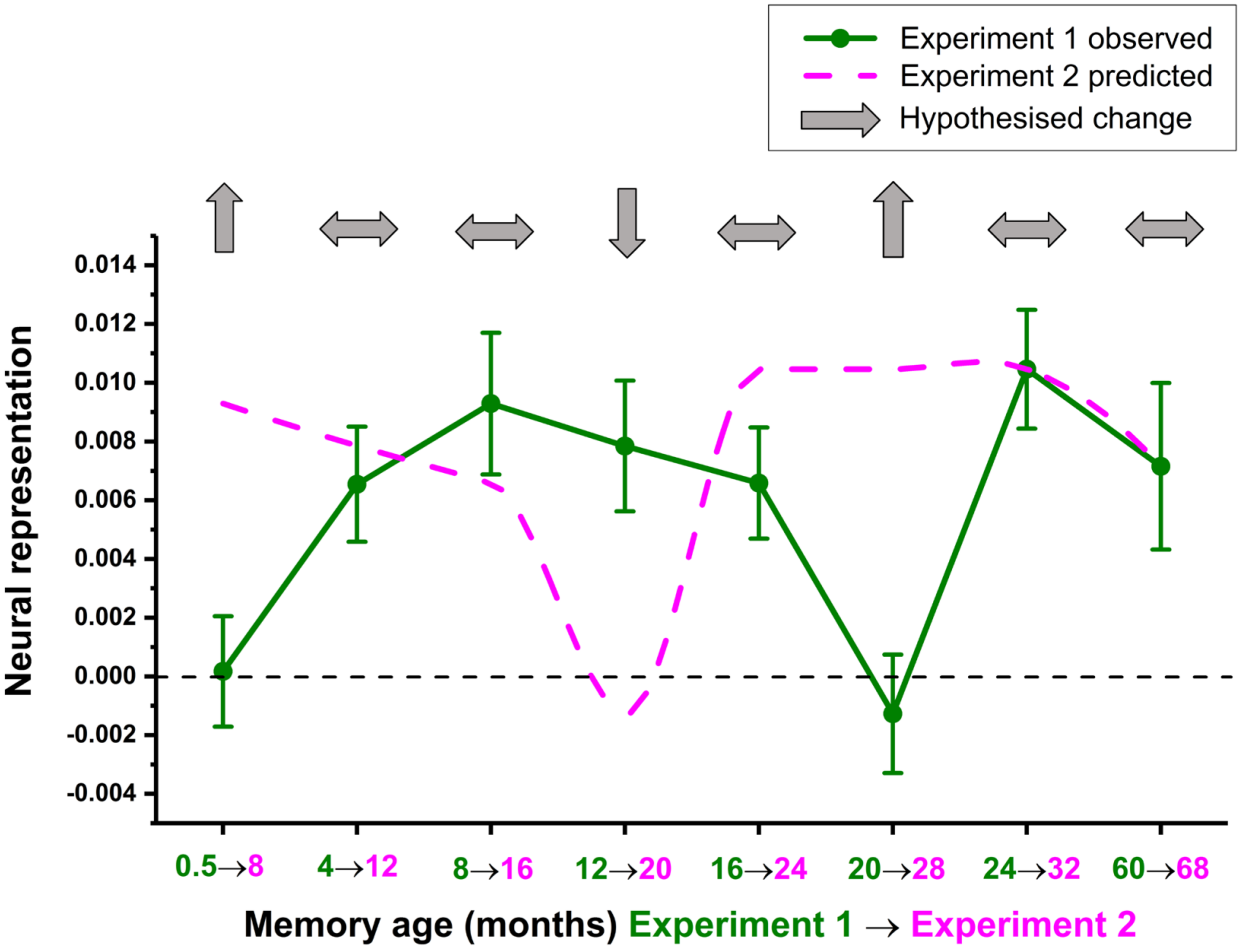
Predicted fMRI changes 8 months later in Experiment 2. Predicted changes in the neural representations of individual autobiographical memories after 8 months (pink dotted line), based on shifting the original observed data forwards by 2 time points for the 16 participants from Experiment 1 (green line) who returned for Experiment 2 (see S4 Data for original and predicted values). Light grey arrows indicate the hypotheses. fMRI, functional magnetic resonance imaging.

Nevertheless, to verify that the results genuinely represented the neural correlates of memory purely as a function of age, one would need to study the effects of the passage of time on the individual neural representations. Therefore, we invited the participants to revisit 8 months later to recall the same memories again both overtly and during scanning; 16 of the participants agreed to return. In order to generate specific predictions for the neural representations during Experiment 2, we took the actual data for the 16 subjects from Experiment 1 who returned 8 months later (Fig 5 green line, in which the nonmonotonic pattern is still clearly evident) and shifted them forwards by 2 time points to simulate the expected pattern 8 months later (Fig 5 pink dotted line). Note that for the 28 M and 32 M time periods in Experiment 2, we assumed they would have the same level of detectability as 24 M old memories, given the absence of data relating to these time periods from Experiment 1. We further assumed the neural representations between 60 M and 68 M would be unchanged.

A comparison of the original and simulated neural representation scores yielded a number of clear hypotheses about how memory representations would change over time in the vmPFC. Two-week-old memories should become detectable 8 months later, while the original 4 M and 8 M old memories should not differ in their representational strength. Twelve-month-old memories from Experiment 1 should be significantly less detectable, whereas 16 M old memories should remain unchanged. The original 20 M old memories should be better represented at 28 M, whereas the 24-and 60-month old-memories from Experiment 1 were not predicted to change over time.

### Experiment 2 (8 months later)

**Fig 6:**
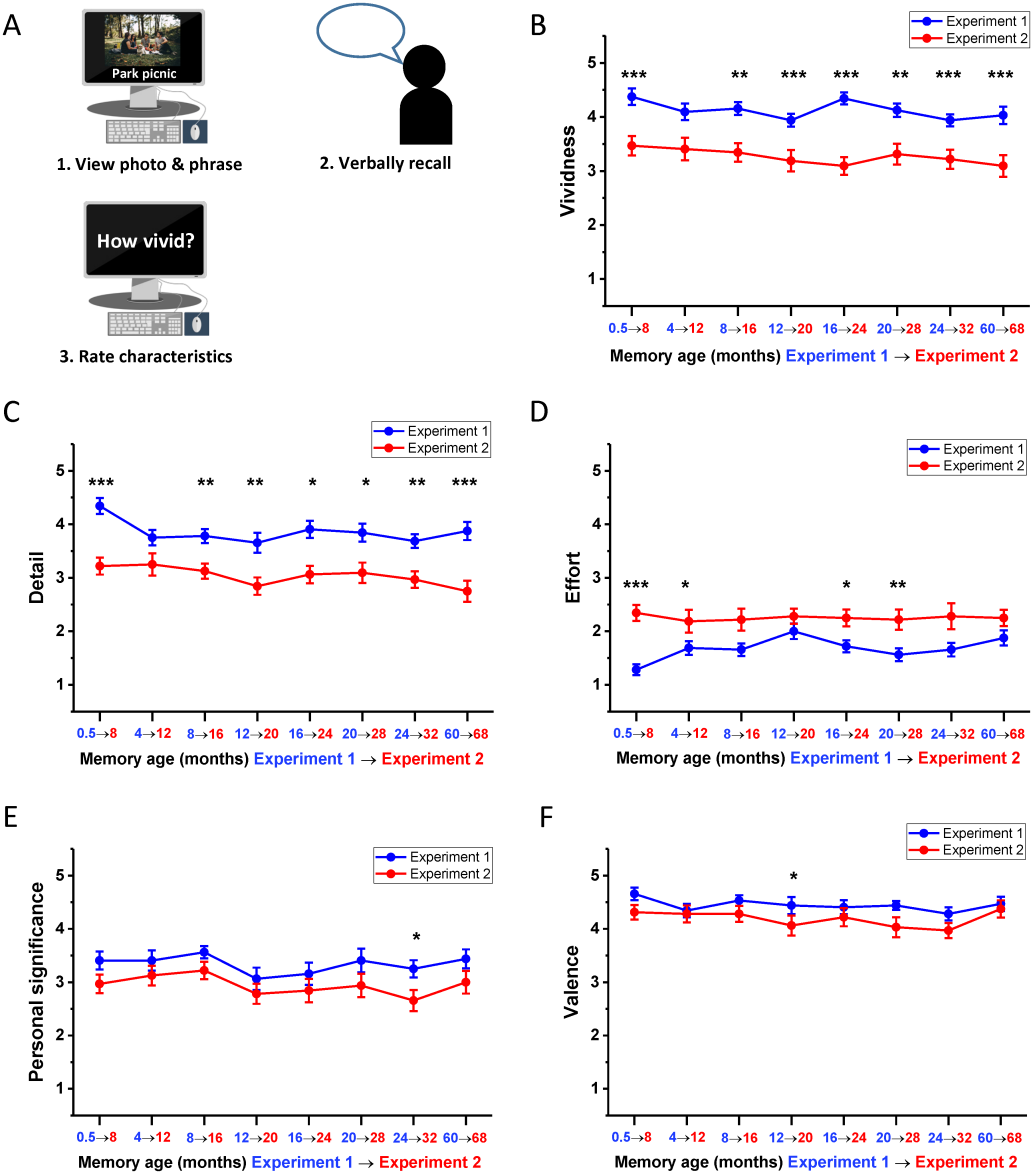
Memory recall and subjective ratings. (A) Schematic of the interview in which participants recalled an autobiographical memory using their previously chosen photograph and cue phrase and rated its characteristics. (B-F) Subjective ratings (means +/− 1 SEM; see also means and SDs in Table A in S1 and S3 Tables) of memory characteristics at each time period for Experiment 1 (blue line, *n* = 16 participants) and how the ratings of the same memories differed 8 months later during Experiment 2 (red line, the same *n* = 16 participants) averaged across the two sets of memories in both cases (see S5 Data for individual ratings across both sets of memories). Ratings were on a scale of 1 to 5, in which 1 was low and 5 was high. For emotional valence: 1–2 = negative, 3 = neutral, 4–5 = positive. Asterisks indicate significant differences in memory ratings between Experiments 1 and 2; * *p* < 0.05, ** *p* < 0.01, *** *p* < 0.001.

One week prior to the fMRI scan, with the assistance of the personal photographs and previously chosen phrases that were used as cues in Experiment 1, the participants verbally recalled and rated the characteristics of their autobiographical memories just as they had done 8 months previously (see Materials and methods and Fig 6A).

#### Subjective ratings of phenomenology remain equivalent across memories

Means and SDs are provided in Table A in S3 Table. Autobiographical memories recalled during Experiment 2 did not differ across time periods on vividness (F_(7,105)_ = 0.83, *p* = 0.564), detail (F_(7,105)_ = 1.30, *p* = 0.257), effort (F_(7,105)_ = 0.11, *p* = 0.998), personal significance (F_(7,105)_ = 1.49, *p* = 0.180), valence (F_(7,105)_ = 1.06, *p* = 0.397), viewpoint (F_(3.42,51.22)_ = 1.24, *p* = 0.31), or motion (F_(3.95,59.32)_ = 1.43, *p* = 0.237). When asked how frequently they had thought about the autobiographical memories in the 8 months between experiments (rated on a 5-point scale from ‘never’ to ‘very frequently’), participants reported some change across time periods (F_(7,105)_ = 3.04, *p* = 0.006). However, the only significant difference between time periods was a lower recall frequency for now 32 M old memories compared to the now 12 M (t_15_ = 3.87, *p* = 0.042). Given the range of responses to this question across conditions (1.50–2.03), clearly, participants had not given the memories much thought in the intervening 8 months. Therefore, all memories recalled in Experiment 2 were extremely well matched in terms of their phenomenology, which reflects the consistency observed in ratings from 8 months onwards in Experiment 1.

There were, however, differences in the absolute values of subjective ratings between the two experiments. There was a decrease in the reported vividness of all memories from Experiment 1 to Experiment 2 (F_(1,15)_ = 88.45, *p* < 0.001), from 0.5 M to when they were 8 M old (t_15_ = 6.21, *p* < 0.001), 8 M to 16 M (t_15_ = 4.21, *p* = 0.006), 12 M to 20 M (t_15_ = 5.48, *p* < 0.001), 16 M to 24 M (t_15_ = 7.07, *p* < 0.001), 20 M to 28 M (t_15_ = 4.10, *p* = 0.008), 24 M to 32 M (t_15_ = 5.97, *p* < 0.001), and 60 M to 68 M (t_15_ = 5.33, *p* < 0.001; Fig 6B). A comparable change was observed in the subjective impression of memory detail recalled following the 8-month interlude (F_(1,15)_ = 126.81, *p* < 0.001), with a drop from 0.5 M to 8 M (t_15_ = 6.26, *p* < 0.001), 8 M to 16 M (t_15_ = 4.03, *p* = 0.009), 12 M to 20 M (t_15_ = 4.78, *p* = 0.002), 16 M to 24 M (t_15_ = 3.72, *p* = 0.016), 20 M to 28 M (t_15_ = 3.67, *p* = 0.018), 24 M to 32 M (t_15_ = 4.55, *p* < 0.003), and 60 M to 68 M (t_15_ = 9.67, *p* < 0.001; Fig 6C). Recalling memories 8 months later was also perceived as more effortful (F_(1,15)_ = 43.32, *p* < 0.001), from 0.5 M to 8 M (t_15_ = −7.81, *p* < 0.001), 4 M to 12 M (t_15_ = −3.30, *p* = 0.039), 16 M to 24 M (t_15_ = −1.95, *p* = 0.021), and 20 M to 28 M (t_15_ = −4.03, *p* = 0.009; Fig 6D). The elapsed time between experiments also led to a reduction in the reported personal significance of memories (F_(1,15)_ = 11.82, *p* = 0.004), from 24 M to 32 M (t_15_ = 3.58, *p* = 0.022; Fig 6E). Ratings of emotional valence also changed over the 8-month period (F_(1,15)_ = 9.78, *p* = 0.007), with a reported attenuation of the positivity of memories from 12 M to 20 M (t_15_ = 3.87, *p* = 0.012; Fig 6F). In addition to these main ratings, no difference was reported in the extent to which memories were recalled from a first- or third-person perspective (F_(1,15)_ = 0.513, *p* = 0.485) over the 8-month period. The extent to which memories were recalled as active or static was altered by the passage of time between experiments (F_(1,15)_ = 11.01, *p* = 0.005), with the original 0.5 M old memories becoming more static when 8 M old (t_15_ = −3.42, *p* = 0.031). See S12 Data for individual ratings for these characteristics.

Despite the observed changes in some subjective ratings from Experiment 1 to Experiment 2, they were unidirectional across all time periods. As such, if the pattern of hypothesised emergence and disappearance of neural representations in vmPFC was to be supported in Experiment 2, then it could not be accounted for by changes in subjective ratings. Additionally, although the changes in subjective ratings across time tend to suggest a comparable degradation in memory quality across all time periods, this may be misleading. The ratings overall were still high, and these absolute changes in values could be influenced by participants’ expectations of their ability to recall memories after an extended period of time with high fidelity, because the objective scoring of memory detail revealed no such pattern, as we report in the next section.

#### A similar level of detail was recalled across experiments

As with Experiment 1, transcripts of participants’ memory interviews during Experiment 2 were scored using the autobiographical interview protocol ([57]; see Materials and methods). A total of 6,444 details were scored (see Table B in S3 Table for means, SD). There was a difference in the number of details recalled across different time periods in Experiment 2 (F_(7,105)_ = 2.49, *p* = 0.021). However, this difference was only observed for external details (F_(7,105)_ = 3.25, *p* = 0.004), with more provided for 28 M memories than 12 M memories (t_15_ = −4.68, *p* = 0.008). As with Experiment 1, the number of internal and external details differed (F_(1,15)_ = 72.57, *p* < 0.001), with more internal details recalled for every time period (all *p* < 0.01). No interaction between time period and type of detail was observed (F_(7,105)_ = 0.87, *p* = 0.530).

**Fig 7:**
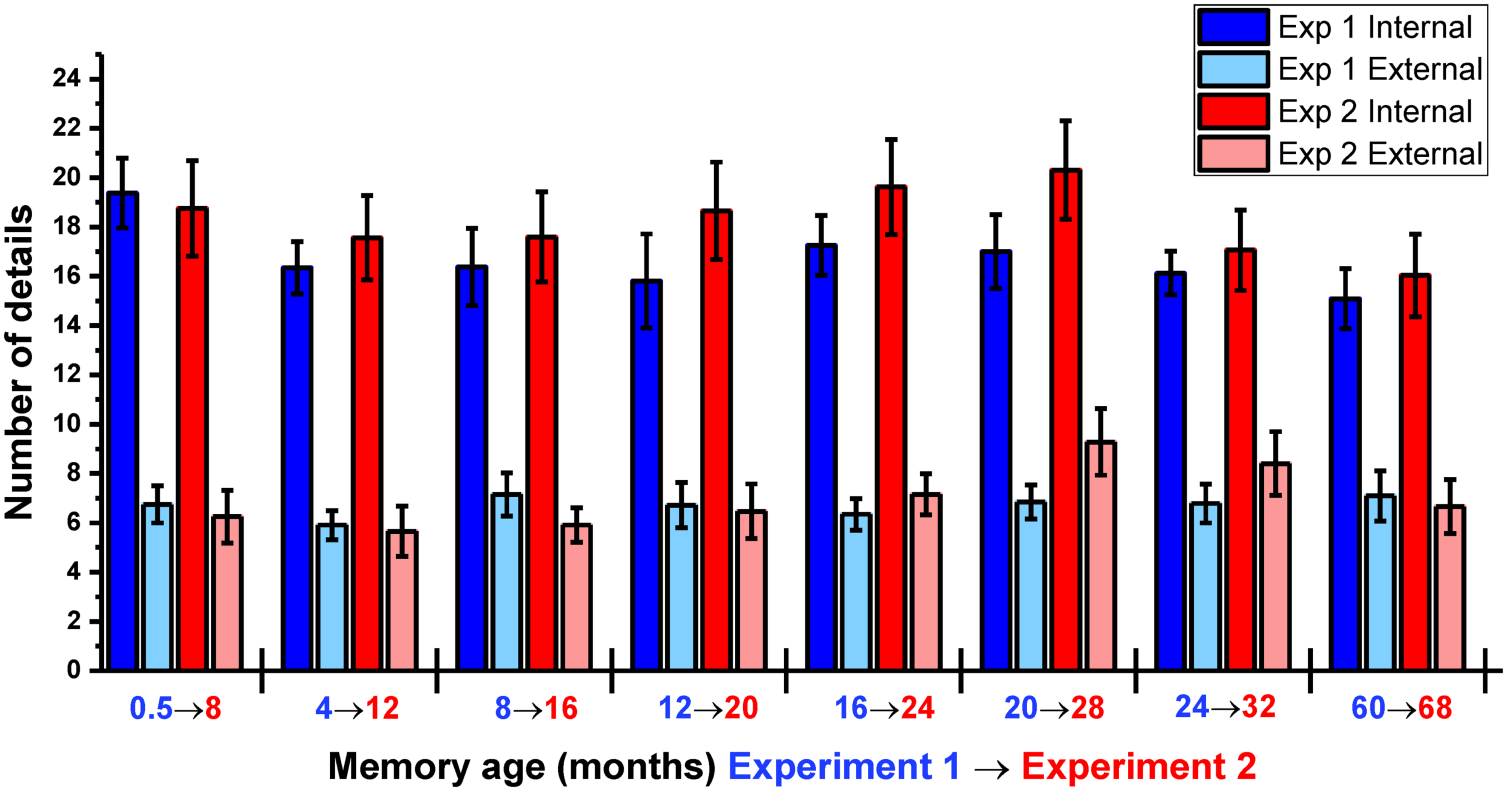
Objective scores for memory details over time. The mean +/− 1 SEM (see also means and SDs in Table B in S1 and S3 Tables) number of internal and external details at each time period for Experiment 1 (blue bars, *n* = 16 participants) and Experiment 2 (red bars, the same *n* = 16 participants), averaged across the two sets of autobiographical memories (see S6 Data for individual participant scores).

When the objective scores for both experiments were compared, no significant difference was observed in the overall number of details provided 8 months later (F_(1,15)_ = 1.93, *p* = 0.185; Fig 7). Furthermore, there was no significant interaction between experiment and time period (F_(1,15)_ = 1.97, *p* = 0.066), indicating that the amount of details provided for memories from any particular time period in Experiment 1 was not affected by the passage of time. Finally, no interaction was observed between experiment and type of detail provided (F_(1,15)_ = 2.27, *p* = 0.153), showing that the ratio of internal to external details was preserved across experiments.

#### vmPFC memory representations undergo the predicted time-dependent changes

Participants were scanned in an identical fashion as Experiment 1 (see Materials and methods and Fig 3B), and neural representation scores for memories from each time point were again calculated.

**Fig 8:**
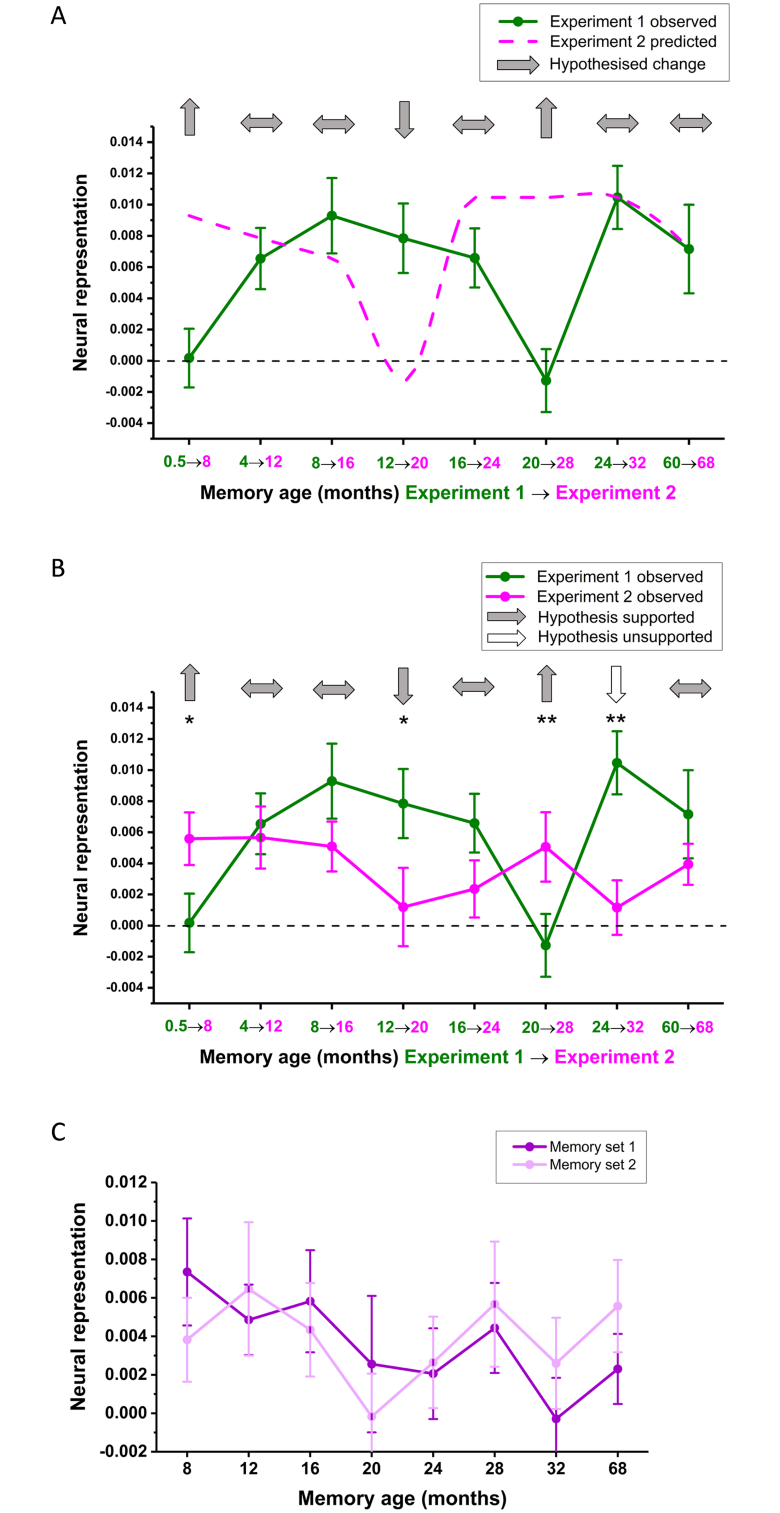
fMRI results of Experiment 2. (A) A reminder of the hypothesised changes in neural representations from Experiment 1 (green line) to Experiment 2 (pink line, reprinted from Fig 5). (B) Mean +/− 1 SEM neural representation scores at each time point averaged across the two sets of memories for Experiment 2 (pink line, *n* = 16 participants) compared to the same memories from 8 months previously (green line, the same *n* = 16 participants). Light grey and white arrows indicate supported and unsupported hypotheses, respectively; * *p* < 0.05, ** *p* < 0.01. (C) Neural representation scores at each time point for Experiment 2, plotted separately for the two sets of autobiographical memories. See S7 Data for individual participant scores. fMRI, functional magnetic resonance imaging.

When comparing the neural representation scores of memories from the eight original time periods in Experiment 1 with those of the same memories 8 months later during Experiment 2, a main effect for experiment (F_(1,15)_ = 2.35, *p* = 0.146) or time period (F_(7,105)_ = 1.18, *p* = 0.323) was not observed; however, an interaction between experiment and time period emerged (F_(7,105)_ = 3.46, *p* = 0.002). Closer examination via our planned comparisons (Fig 8A) revealed that 7 out of the 8 predictions made on the basis of the Experiment 1 findings were supported (Fig 8B). The original 0.5 M old memories had increased in their representational strength in vmPFC 8 months later (t_15_ = −1.84, *p* = 0.043), while the neural representation scores of the 4 M and 8 M old memories were essentially unchanged at 12 M (t_15_ = 0.43, *p* = 0.677) and 16 M (t_15_ = 1.22, *p* = 0.242), respectively. As expected, the original 12 M old memories from Experiment 1 were, 8 months later, more poorly represented in vmPFC when 20 M old (t_15_ = 1.85, *p* = 0.042). The original 16 M old memories were unchanged in their representational strength at 24 M (t_15_ = 1.38, *p* = 0.187), while 20 M old memories were significantly more detectable in vmPFC at 28 M (t_15_ = −2.69, *p* = 0.008). The most remote 60 M memories did not differ in their neural representation scores 8 months later (t_15_ = 0.86, *p* = 0.402). In fact, the only finding that was inconsistent with the predictions generated by Experiment 1 was a decrease in the representation of 24 M old memories when they were 32 M of age (t_15_ = −2.69, *p* = 0.009). However, this prediction was based on the assumption that memories do not undergo further dynamic shifts in neural representation between 2 and 5 years, which may not be the case, and we did not have 32 M data from Experiment 1 to corroborate this finding.

For completeness, Fig 8C plots the neural representation scores for the two sets of memories in Experiment 2. As previously observed in Experiment 1, the two sets of memories displayed a similar time course in terms of their neural representations, despite being recalled in separate scanning sessions in a randomised order and analysed separately. As with Experiment 1, when examining other brain areas within the partial volume in Experiment 2, in no case did we find a significant difference in memory detectability across time periods.

Following scanning in Experiment 2, participants completed 3 additional ratings. They were asked to indicate the extent to which the memories were changed by the six repetitions during scanning on a scale ranging from 1 (not at all) to 5 (completely). As in Experiment 1, they reported that the memories were not changed very much by repetition (mean: 2.56, SD: 0.81). They were also asked how often they thought of the experience of recalling the memories in Experiment 1 while performing the scanning task in Experiment 2 on a scale of 1 (not at all) to 5 (during every memory). Participants indicated they rarely thought about Experiment 1 (mean: 1.75, SD: 0.93). Finally, the consistency of recall across time periods during the scanning session did not differ in Experiment 2 (F_(7,105)_ = 0.59, *p* = 0.761) or between the two experiments (F_(1,15)_ = 0.12, *p* = 0.733; see also Table A in S1 and S3 Tables).

## Discussion

This study exploited the sensitivity of RSA to detect not only the extent to which memories of different ages were represented in the vmPFC but how these representations changed over time. During Experiment 1, we observed detectability in vmPFC for memories at 4 M to 12 M of age, which was also evident at 24 M and 60 M. As expected, recent 0.5 M old memories were poorly represented in vmPFC in comparison. Curiously, however, the same lack of detectability in vmPFC was observed for memories that were 16 M to 20 M old. This pattern persisted across separate sets of memories and was replicated in a follow-up study 8 months later with the same participants and memories. Behavioural data failed to account for these time-dependent representational changes in either experiment, and other regions failed to show a significant change in memory representations over time. These findings are difficult to accommodate within any single theoretical account of long-term memory consolidation [9, 12, 61–63], as neocortical recruitment is generally assumed to involve an ascending linear trajectory. Consolidation has been characterised as fluid and continuous [64], but the nonmonotonic vmPFC engagement observed here suggests additional complexity in its temporal recruitment.

### Possible mechanisms underlying nonmonotonic vmPFC recruitment

Over the course of consolidation in this study, the vmPFC twice alternated between disengagement and engagement, indicative of 4 separate stages. Below we consider, based on the latest theoretical developments and empirical research on systems-level consolidation and vmPFC functioning, the time-dependent processes which could underlie such a nonmonotonic pattern.

#### Less than 1 month: Quiescence during the early stages of systems-level consolidation

During retrieval of memories less than 1 month of age, there was a notable absence of vmPFC recruitment. This is consistent with previous human studies showing weaker representations of recent memories, using pattern classification [54], and lower overall levels of fMRI activity [41]. Similarly, in animals, prefrontal activation is reduced during recent memory recall [18, 19], with lesions of this region generally preserving recent memory retrieval [21, 24]. Although prefrontal cells may be ‘tagged’ for subsequent consolidation around the time of encoding [14], they remain functionally immature and do not appear to significantly contribute to memory retrieval at this stage [26].

#### Four months to 1 year: Disambiguation of competing consolidated representations

Over the subsequent three time periods in this study, vmPFC memory representations progressively strengthened. This echoes the time-dependent increases in prefrontal cortex activity observed in animal studies during memory retrieval [19, 65], and the disruption of remote memory following prefrontal inactivation [19, 21, 24]. While it has been demonstrated that the interim replay of recent experiences in the prefrontal cortex [66] coincides with lasting structural changes that facilitate subsequent recall [29], it is still unclear how these consolidated representations are utilised during retrieval. One prominent hypothesis is that the prefrontal cortex suppresses irrelevant representations [67], with corresponding evidence in animals that context-inappropriate memories are triggered following prefrontal inactivation [68]. Similarly, in humans, vmPFC damage impairs the ability to suppress inappropriate memories through confabulation [34] and produces a tendency to confuse memories that have taken place in different contexts [69]. This is of potential relevance to the 4- to 12-month time period identified in the current study, as people remember vastly more memories from the past year than more remote life periods [70]. Therefore, the demands on memory disambiguation (the ability to correctly select from among similar memories during retrieval) are significantly increased across this timescale. For example, in attempting to recall the specific events from a party one attended months previously, multiple contemporaneous experiences that involve the same people or have taken place in a similar context could interfere with recollection. While humans possess a large capacity for real-world stimuli in recent memory, an abundance of stored competing representations can be detrimental to memory performance during retrieval [71]. Therefore, vmPFC recruitment at these time points may reflect a suppression of distractor representations that are inappropriate to the current retrieval intentions. Importantly, this would be a memory-specific process that would generate a consistent neural pattern every time a particular experience is recalled.

#### Sixteen to 20 months: Time-induced decay negates the need for disambiguation

The progressive vmPFC disengagement observed over the following 8 months suggests the suppression of interfering memories becomes less of a necessity over this period. Forgetting is a key attribute of an optimally functioning memory system [72], and the number of autobiographical events individuals can recall has been shown to decrease substantially between 1 and 2 years before levelling off [73]. Therefore, the reduction in availability of potentially interfering memories from this time period may relieve the vmPFC from its role in disambiguating them from memories that have persisted through the consolidation process. For example, one may return from a vacation with many memories that contain multiple overlapping features, but this will inevitably be reduced to a few distinct experiences as time goes on.

#### Two years to 5 years: The emergence of schematic representations

If disambiguation ceases to be an issue for older memories, the robust reengagement of vmPFC at more remote time periods suggests locally consolidated representations come to be used in a different way to assist in recall. From a theoretical perspective, systems-level consolidation is no longer viewed as the time-limited stabilisation of a static engram. Rather, the passage of time and repeated retrieval is thought to generate an additional representation that can complement the original detailed memory [10]. This emergent representation is schematic in nature, with an emphasis on general rather than specific details, and forms part of a flexible memory system that adapts to current demands. The network hub that supports schematic representations, suggested by both animal [17] and human neuroimaging [74] studies, is the medial prefrontal cortex. Further evidence is provided by patients with vmPFC lesions, who are resistant to false memory effects because schemas that would conflate actually studied and similar unseen words are not activated during retrieval [75]. Therefore, it is likely that the nature of memory representations in the vmPFC transform over the course of consolidation to become more schematic in nature. Accordingly, the reengagement of vmPFC activity at more remote time points in this study could point to the deployment of memory-congruent schema to assist in retrieval. For example, the vivid recollection of a memory from 5 years previously will likely require reorientation to an increasingly unfamiliar environment, an altered social network, and a different personal mindset. This may be facilitated by a rapid instantiation of relevant schematic representations in the vmPFC to bias retrieval in posterior brain regions, as proposed by Gilboa and Marlatte [76]. The nonmonotonic recruitment observed here may, therefore, reflect not just the consolidation of neural representations but their evolution over time and, most importantly, the way in which they are used to facilitate precise and holistic recollection. vmPFC engagement during recall likely reflects not just task-related recruitment but also communication with the hippocampus and other neocortical regions.

### Relevance to systems-level consolidation theories

The current findings have potential implications for the two dominant theoretical perspectives on systems-level consolidation. Standard consolidation theory [7] predicts that the passage of time promotes the strengthening of neural representations in the neocortex, but the duration of this process in humans is poorly specified. The current results suggest this process is accomplished over a relatively fast timescale on the order of months. The alternative perspective on consolidation, multiple trace theory and transformation hypothesis [10], posits that over time, consolidation promotes the emergence of schematic, gist-like representations in the neocortex, which complement the original detailed memory. The reengagement of the vmPFC at 2 years in this study may reflect the emergence of these generalised representations to facilitate specific recall at more remote time points. Therefore, the consolidation of new memories in the neocortex may be reasonably rapid, whereas the transformation of these engrams may take place over a much longer timescale.

Using an autobiographical memory paradigm to study consolidation is preferable to laboratory-based episodic memory tests by virtue of its ecological validity, availability of temporally distant stimuli, clinical significance, and context-dependent equivalence to animal tasks. However, studying autobiographical memory carries with it potential confounds that can affect interpretation of results. In the sections that follow, we consider why these factors cannot account for our observations.

### Consistency of recall and forgetting

Older memories may yield a higher RSA score if they are more consistently recalled. Here, however, participants actually rated 0.5 M memories as more consistently recalled than 60-month-old memories. Older memories were not impoverished in detail when compared to the detail available for recent memories. Moreover, an inspection of interview transcripts across experiments revealed participants rarely offered new details for previous memories when retested, countering the suggestion that increased detectability of old memories may arise from the insertion of new episodic or semantic details [77]. The consistency in recalled detail across experiments could be attributable to participants recalling in Experiment 2 what they had said during Experiment 1. However, whether or not participants remembered by proxy is irrelevant, as they still recalled the specific details of the original event, removing forgetting as a potential explanation of changes in neural patterns over time.

### The influence of repetition

Retrieving a memory initiates reconsolidation, a transient state in which memories are vulnerable to interference [78, 79]. Therefore, repeated retrieval may cause this process to have an influence on neural representations. However, all memories were recalled 1 week before the fMRI scan, so if such an effect was present, it would be matched across time points. Retrieval at this stage may also accelerate consolidation [80], yet if this were a major influence, we would likely have found 0.5 M memories to be more detectable than they were. Further repetition of memories within the scanner in Experiment 1 took place over a timescale that could not affect consolidation processes or interpretation of the initial neural data. Nevertheless, this could arguably affect vmPFC engagement over a longer period of time [81] and thus perturb the natural course of consolidation, influencing the results of Experiment 2. However, given that 7 out of the 8 specifically hypothesised temporally sensitive changes in neural representations were supported, an altered or accelerated consolidation time course appears highly unlikely. Again, recall recency was matched in Experiment 2 by the memory interview, and recall frequency between experiments was low.

Taking a more general and parsimonious perspective, the ratings demonstrate that, naturally, all memories are recalled on an occasional basis (Table A in S1 Table); therefore, it seems highly unlikely that a mere six repetitions within a scanning session would significantly alter the time course of systems-level consolidation. It should also be noted that successful detection of neural patterns relied on the specific content of each memory rather than being due to generic time-related retrieval processes (S4 Fig). One alternative to the current two-experiment longitudinal design to limit repetition across experiments would be to have a different group of participants with different memories for the second experiment. However, the strength of the current approach was the ability to track the transformation in neural patterns of the same memories over time.

### The effect of selection

An alternative interpretation of the time-sensitive vmPFC engagement is a systematic bias in the content of selected memories—for example, annual events coinciding across all participants, such as a seasonal holiday. However, recruitment took place over a period of 5 months in an evenly spaced manner, ensuring that such events did not fall into the same temporal windows across participants. The occurrence of personal events such as birthdays was also random across participants. The use of personal photographs as memory cues also limited the reliance on time of year as a method for strategically retrieving memories. Furthermore, the nature of memory sampling was that unique, rather than generic, events were eligible, reducing the likelihood of events that were repeated annually being included. Memory detectability was high at 12-month intervals such as 1, 2, and 5 years in this study, suggesting perhaps it is easier to recall events that have taken place at a similar time of year to the present. However, this should have been reflected in behavioural ratings and equivalently strong neural representations for recent memories, but neither was observed. Most importantly, if content rather than time-related consolidation was the main influence on memory detectability, then we would not have observed any change in neural representation scores from Experiment 1 to Experiment 2, rather than the hypothesised shifts that emerged.

A related concern is that memories across time differ in nature because they differ in availability. Successful memory search is biased towards recency, meaning there are more events to choose from in the last few weeks than in remote time periods. Here, this confound is circumvented by design, given that search was equivalently constrained and facilitated at each time point by the frequency at which participants took photographs, which was not assumed to change in a major way over time. These enduring ‘snap-shots’ of memory, located within tight temporal windows (see Materials and methods), meant that memory selection was not confounded by retrieval difficulty or availability. It could also be argued that selection of time points for this study should have been biased towards recency, given that most forgetting occurs in the weeks and months after learning. However, it is important to dissociate systems-level consolidation from forgetting, as they are separate processes that are assumed to follow different time courses. Memory forgetting follows an exponential decay [82], whereas systems-level consolidation has generally been assumed, until now, to be gradual and linear [83]. Our study was concerned only with vivid, unique memories that were likely to persist through the systems-level consolidation process.

A further potential concern regarding memory selection is that recent and remote memories that are comprised of equivalent levels of detail must be qualitatively different in some way. For example, selected remote memories must have been highly salient at the time of encoding to retain such high levels of detail. However, the underlying assumption that individual memories invariably become detail impoverished over time does not necessarily hold. While the volume of memories one can recall decreases over time [84], the amount of details one can recall from individual consolidated memories can actually increase over a 1-year delay [85]. While generalised representations are thought to emerge over the course of consolidation, they do not necessarily replace the original detailed memories [10], and the equivalent level of detail provided by participants across the two experiments here would suggest that memory specificity can be preserved over time. Furthermore, the possibility that remote memory selection may still be biased towards more salient memories is rendered unlikely by the method of memory sampling employed here. Because memories were chosen only from available photographic cues, the salience of recent and remote events was determined at the time of taking the photograph and not during experimentation. These photographs served as potent triggers of remote memories that were not necessarily more salient than recent memories and that may not have otherwise come to mind using a free-recall paradigm. In addition, one would expect more salient remote memories to score higher than recent memories on subjective ratings of vividness, personal significance, or valence, but this was not the case. Therefore, stronger neural representations at more remote time points were likely due to consolidation-related processes rather than qualitative difference between recent and remote experiences at the time of encoding.

### Value

Given that the medial prefrontal cortex is often associated with value and emotional processing [86], could these factors have influenced the current findings? Humans display a bias towards consolidating positive memories [87], and remembered information is more likely to be valued than that which is forgotten [88]. Activity in vmPFC during autobiographical memory recall has been found to be modulated by both the personal significance and emotional content of memories [89]. However, in the current two experiments, memories were matched across time periods on these variables, and the selection of memories through photographs taken on a day-to-day basis also mitigated against this effect. In the 8 months between experiments, memories either remained unchanged or decreased slightly in their subjective ratings of significance and positivity, suggesting that these factors are an unlikely driving force behind the observed remote memory representations in vmPFC. For example, if recent memories in Experiment 1 were not well represented in vmPFC because they were relatively insignificant, there is no reason to expect them to be more so 8 months later, yet their neural representation strengthened over time nonetheless.

### Relation to previous findings

A methodological discrepancy between this experiment and that conducted by Bonnici and colleagues [54] is the additional use of a photograph to assist in cueing memories. One possible interpretation of the neural representation scores is they represent a role for the vmPFC in the maintenance of visual working memory following cue offset. However, the prefrontal cortex is unlikely to contribute to maintenance of visual information [90]. Furthermore, if this was the driving effect behind neural representations here, the effect would be equivalent across time periods, yet it was not.

There is, however, an obvious inconsistency between the findings of the current study and that of Bonnici and colleagues [54]. Unlike that study, we did not detect representations of 0.5 M old memories in vmPFC. It could be that the support vector machine classification–based MVPA used by Bonnici and colleagues [54] is more sensitive to detection of memory representations than RSA; however, the current study was not optimised for such an analysis, because it necessitated an increased ratio of conditions to trials. Nonetheless, the increase in memory representation scores from recent to remote memories was replicated and additionally refined in the current study with superior temporal precision. One observation that was consistent with the Bonnici findings was the detection of remote memories in the hippocampus, which also supports theories positing a perpetual role for this region in the vivid retrieval of autobiographical memories [10, 12]. However, the weak detectability observed at more recent time points may reflect a limitation of the RSA approach employed here to detect sparsely encoded hippocampal patterns, which may be overcome by a more targeted subfield analysis [91].

There are, however, distinct advantages to the use of RSA over pattern-classification MVPA. RSA is optimal for a condition-rich design, as it allows for the relationships between many conditions to be observed. For example, in the current experiment, a visual inspection of the group RSA matrix (S1 Fig) does not reveal an obvious clustering of recent or remote memories that would indicate content-independent neural patterns related to general retrieval processes. The approach employed by Bonnici and colleagues [54] assessed the distinctiveness of memories within each time point from each other in order to detect memory representations. Should the neural patterns of a single memory become more consistent over time, yet also more similar to memories of the same age because of generic time-dependent mechanisms of retrieval, pattern classification would fail to detect a representation when one is present. In the current study, however, the two can be assessed separately, revealing memories at each time point become distinct from both memories of all other ages (Fig 4A) and identically aged memories (Fig 4C). The machine-learning approach employed by Bonnici and colleagues [54] to decode memory representations also requires the division of data into ‘training’ and ‘testing’ sets to classify unseen neural patterns [53]. This reduces the number of trials available for analysis, which would have been suboptimal for the current design because it would have necessitated an increased number of conditions and fewer trials per memory, whereas this restriction is not a necessity for RSA. Finally, because the pattern classification approach used by Bonnici and colleagues [54] compared memories from each time point directly to each other, they could not be analysed independently. In the current RSA design, the two sets of memories could be analysed separately from each other to ascertain if the temporal patterns could be replicated in an independent set of data. As is evident in Fig 4B, the nonmonotonic pattern of vmPFC recruitment was present in both sets of memories. The suitability of each MVPA method, therefore, depends on the study design and the research questions being posed.

In the light of our hypotheses, Experiment 2 generated one anomalous finding. Twenty-four-month-old memories from Experiment 1 were no longer well represented 8 months later. Why memories around 32 M of age are not as reliant on vmPFC is unclear, but unlike other time periods, we cannot verify this finding in the current experiment, as we did not sample 32 M memories during Experiment 1.

### Summary

The current results revealed that the recruitment of the vmPFC during the expression of autobiographical memories depends on the exact stage of systems-level consolidation and that retrieval involves multiple sequential time-sensitive processes. These temporal patterns were remarkably preserved across completely different sets of memories in one experiment and closely replicated in a subsequent longitudinal experiment with the same participants and memories. These findings support the notion that the vmPFC becomes increasingly important over time for the retrieval of remote memories. Two particularly novel findings emerged. First, this process occurs relatively quickly, by 4 months following an experience. Second, vmPFC involvement after this time fluctuates in a highly consistent manner, depending on the precise age of the memory in question. Further work is clearly needed to explore the implications of these novel results. Overall, we conclude that our vmPFC findings may be explained by a dynamic interaction between the changing strength of a memory trace, the availability of temporally adjacent memories, and the concomitant differential strategies and schemas that are deployed to support the successful recollection of past experiences.

## Materials and methods

### Ethics statement

This study was approved by the local research ethics committee (University College London Research Ethics Committee, approval reference 6743/002). All investigations were conducted according to the principles expressed in the Declaration of Helsinki. Written informed consent was obtained for each participant.

### Experiment 1

#### Participants

Thirty healthy, right-handed participants (23 female) took part (mean age 25.3, SD 3.5, range 21–32). All had normal or corrected-to-normal vision.

#### Memory interview and selection of autobiographical memories

Participants were instructed to select at least 3 photographs from each of 8 time points in their past (0.5 M, 4 M, 8 M, 12 M, 16 M, 20 M, 24 M, and 60 M relative to the time of taking part in the experiment) that reminded them of vivid, unique, and specific autobiographical events. The sampling was retrospective in that the photographs were chosen from the participants’ preexisting photograph collections and not prospectively taken with the study in mind. Highly personal, emotionally negative, or repetitive events were deemed unsuitable. An additional requirement was that memories from the same time period should be dissimilar in content. For the four most recent time periods (0.5 M–12 M), the memories should have taken place within a temporal window of 2 weeks on either side of the specified date, yielding a potential window of 1 month; for the next three time points (16M-24M), 3 weeks on either side to allow a window of 6 weeks; and 1 month on either side for the most remote time point (60 M), giving a 2-month window. This graded approach was adopted to balance temporal precision with the availability of suitable memories at more remote time points.

Participants were asked to describe in as much detail as possible the specific autobiographical memory elicited by a photograph. General probes were given by the interviewer when appropriate (e.g., ‘what else can you remember about this event?’). Participants were also asked to identify the most memorable part of the event that took place within a narrow temporal window and unfolded in an event-like way. They then created a short phrase pertaining to this episode, which was paired with the photograph to facilitate recall during the subsequent fMRI scan (Fig 1A). Participants were asked to rate each memory on a number of characteristics (see main text, Figs 1 and 6, S1 and S3 Tables), and 2 memories from each time period that satisfied the criteria of high vividness and detail and ease of recall were selected for recollection during the fMRI scan.

#### Behavioural analyses

The interview was recorded and transcribed to facilitate an objective analysis of the details, and the widely used autobiographical interview method was employed for scoring [57]. Details provided for each memory were scored as either ‘internal’ (specific events, temporal references, places, perceptual observations, and thoughts or emotions) or ‘external’ (unrelated events, semantic knowledge, repetition of details, or other more general statements). To assess interrater reliability, a subset of 16 memories (*n* = 2 per time period) were randomly selected across 16 different subjects and scored by another experimenter blind to the aims and conditions of the study. Intraclass coefficient estimates were calculated using SPSS statistical package version 22 (SPSS, Chicago, IL) based on a single-measure, absolute-agreement, two-way random-effects model.

As 2 memories per time period were selected for later recall in the scanner, behavioural ratings were averaged to produce 1 score per time period. Differences in subjective memory ratings across time periods were analysed using a one-way repeated-measures ANOVA with Bonferroni-corrected paired *t* tests. Differences in objective memory scores of internal and external details across time periods were analysed using a two-way repeated-measures ANOVA with Bonferroni-corrected paired *t* tests. A threshold of *p* < 0.05 was used throughout both experiments. All ANOVAs were subjected to Greenhouse-Geisser adjustment to the degrees of freedom if Mauchly’s sphericity test identified that sphericity had been violated.

#### Task during fMRI scanning

Participants returned approximately 1 week later (mean 6.9 days, SD 1) to recall the memories while undergoing an fMRI scan. Prior to the scan, participants were trained to recall each of the 16 memories within a 12-second recall period (as in Bonnici and colleagues [54], Bonnici and Maguire [55]) when cued by the photograph alongside its associated cue phrase. There were 2 training trials per memory, and participants were asked to vividly and consistently recall a particular period of the original event that unfolded across a temporal window matching the recall period.

During scanning, participants recalled each memory 6 times (6 trials × 16 memories = 96 trials). The two memories from each time period were never recalled together in the same session, nor was any one memory repeated within each session, resulting in 12 separate short sessions with 8 trials in each, an approach recommended for optimal detection of condition-related activity patterns using MVPA [92]. Trials were presented in a random order within each session. On each trial, the photograph and associated preselected cue phrase relating to each event were displayed on screen for 3 seconds. Following removal of this cue, participants then closed their eyes and recalled the memory. After 12 seconds, the black screen flashed white twice to cue the participant to open their eyes. The participant was then asked to rate how vivid the memory recall had been, using a 5-key button box, on a scale of 1–5, in which 1 was not vivid at all, and 5 was highly vivid. When the least vivid trials were excluded, the mean number of trials (/6) selected for analysis from each time point was as follows: 0.5 M: 5.65 (SD 0.57), 4 M: 5.50 (SD 0.56), 8 M: 5.43 (SD 0.55), 12 M: 5.50 (SD 0.63), 16 M: 5.50 (SD 0.59), 20 M: 5.43 (SD 0.65), 24 M: 5.42 (SD 0.56), 60 M: 5.23 (SD 0.69).

#### MRI data acquisition

Structural and functional data were acquired using a 3T MRI system (Magnetom TIM Trio, Siemens Healthcare, Erlangen, Germany). Both types of scan were performed within a partial volume that incorporated the entire extent of the vmPFC (Fig 3A).

Structural images were collected using a single-slab 3D T2-weighted turbo spin echo sequence with variable flip angles (SPACE) [93] in combination with parallel imaging to simultaneously achieve a high image resolution of approximately 500 μm, high sampling efficiency, and short scan time while maintaining a sufficient signal-to-noise ratio (SNR). After excitation of a single axial slab, the image was read out with the following parameters: resolution = 0.52 × 0.52 × 0.5 mm, matrix = 384 × 328, partitions = 104, partition thickness = 0.5 mm, partition oversampling = 15.4%, field of view = 200 × 171 mm, echo time (TE) = 353 ms, TR = 3,200 ms, GRAPPA × 2 in phase-encoding (PE) direction, bandwidth = 434 Hz/pixel, echo spacing = 4.98 ms, turbo factor in PE direction = 177, echo train duration = 881, averages = 1.9. For reduction of signal bias due to, for example, spatial variation in coil sensitivity profiles, the images were normalized using a prescan, and a weak intensity filter was applied as implemented by the scanner’s manufacturer. To improve the SNR of the anatomical image, 3 scans were acquired for each participant, coregistered, and averaged. Additionally, a whole-brain 3D FLASH structural scan was acquired with a resolution of 1 × 1 × 1 mm.

Functional data were acquired using a 3D echo planar imaging (EPI) sequence, which has been demonstrated to yield improved BOLD sensitivity compared with 2D EPI acquisitions [94]. Image resolution was 1.5 × 1.5 × 1.5 mm, and the field of view was 192 mm in-plane. Forty slices were acquired with 20% oversampling to avoid wrap-around artefacts due to imperfect slab excitation profile. The TE was 37.30 ms, and the TR was 3.65 seconds. Parallel imaging with GRAPPA image reconstruction [95] acceleration factor 2 along the PE direction was used to minimize image distortions and yield optimal BOLD sensitivity. The dummy volumes necessary to reach steady state and the GRAPPA reconstruction kernel were acquired prior to the acquisition of the image data, as described by Lutti and colleagues [94]. Correction of the distortions in the EPI images was implemented using B0-field maps obtained from double-echo FLASH acquisitions (matrix size 64 × 64; 64 slices; spatial resolution 3 × 3 × 3 mm; short TE = 10 ms; long TE = 12.46 ms; TR = 1,020 ms) and processed using the FieldMap toolbox available in SPM [96].

#### MRI data preprocessing

fMRI data were analysed using SPM12 (www.fil.ion.ucl.ac.uk/spm). All images were first bias corrected to compensate for image inhomogeneity associated with the 32-channel head coil [97]. Field maps collected during the scan were used to generate voxel displacement maps. EPIs for each of the 12 sessions were then realigned to the first image and unwarped using the voxel displacement maps calculated above. The three high-resolution structural images were averaged to reduce noise and coregistered to the whole-brain structural scan. EPIs were also coregistered to the whole-brain structural scan. Manual segmentation of the vmPFC was performed using ITK-SNAP on the group-averaged structural scan normalised to MNI space. The normalised group mask was warped back into each participant’s native space using the inverse deformation field generated by individual-participant structural scan segmentations. The overlapping voxels between this participant-specific vmPFC mask and the grey matter mask generated by the structural scan segmentation were used to create a native-space grey matter vmPFC mask for each individual participant.

#### RSA

Functional data were analysed at the single-subject level without warping or smoothing. Each recall trial was modelled as a separate GLM, which comprised the 12-second period from the offset of the memory cue to just before the white flash that indicated to the participant they should open their eyes. Motion parameters were included as regressors of no interest. RSA [56] was performed using the RSA toolbox (http://www.mrc-cbu.cam.ac.uk/methods-and-resources/toolboxes/) and custom MATLAB (version R2014a) scripts. In order to account for the varying levels of noise across voxels, which can affect the results of multivariate fMRI analyses, multivariate noise normalisation [98] was performed on the estimated pattern of neural activity separately for each trial. This approach normalises the estimated beta weight of each voxel using the residuals of the first-level GLM and the covariance structure of this noise. This results in the down-weighting of noisier voxels and a more accurate estimate of the task-related activity of each voxel.

The average number of voxels analysed in the vmPFC across the two sets of memories was 5,252 (SD 1227). Whole ROI-based analysis was preferred to a searchlight approach, which would involve comparing neural with model similarity matrices [99], as we did not have strong a priori hypothesis about changes in neural representations over time against which to test the neural data, nor did we want to make assumptions regarding the spatial distribution of informative voxels in the vmPFC.

As participants recalled 2 memories per time point, the dataset was first split into 2 sets of 8 time points, which were analysed separately using RSA. To characterise the strength of memory representations in the vmPFC, the similarity of neural patterns across recall trials of the same memory was first calculated using the Pearson product-moment correlation coefficient, resulting in a ‘within-memory’ similarity score. Then, the neural patterns of each memory were correlated with those of all other memories, yielding a ‘between-memory’ similarity score. Both within- and between-memory correlations were performed on trials from separate runs. For each memory, the between-memory score was then subtracted from the within-memory score to provide a neural representation score (Fig 3C). This score was then averaged across the two memories at each time point. Results for the left and the right hemispheres were highly similar; therefore, the data we report here are from the vmPFC bilaterally. A distinctive neural pattern associated with the recall of memories at each time period would yield a score significantly higher than 0, which was assessed using a one-sample *t* test. Strengthening or weakening of memory representations as a function of remoteness would result in a significant difference in memory representation scores across time periods, and this was assessed using a one-way repeated-measures ANOVA with post hoc two-tailed paired *t* tests. Error bars on graphs displaying neural representation scores were normalised to reflect within- rather than between-subject variability in absolute values, using the method recommended by Cousineau [100] for within-subjects designs. The range of values that we observed are entirely consistent with those in other studies employing a similar RSA approach in a variety of learning, memory, and navigation tasks in a wide range of brain regions [101–110].

#### Searchlight analysis

An RSA searchlight analysis was conducted in normalised space on multivariate noise-normalised data within the ROI. This approach selected every voxel within the ROI and, using a volumetric approach, which is constrained by the shape of the ROI, expanded the area around that voxel until an area of 160 voxels was reached. Within each of these spheres, memories were correlated with themselves and other memories, analogous to the standard ROI approach. Then, the resulting neural RSM was correlated using Spearman’s rank correlation coefficient with a model RSM that consisted of ones along the diagonal and zeros on the off-diagonal. This model RSM was used to detect if individual memories were detectable across all time points. For every voxel, the average correlation from every sphere it participated in was calculated to generate a more representative score of its informational content. Parametric assumptions regarding the spatial distribution of unsmoothed data may not hold. Therefore, we used statistical nonparametric mapping (SnPM13) on the resulting searchlight images. We used 10,000 random permutations, a voxel-level significance threshold of t = 3, and a family-wise error–corrected cluster-wise threshold of *p* < 0.05 within an ROI.

### Experiment 2

#### Participants

Sixteen of the 30 participants who took part in Experiment 1 returned to take part in Experiment 2 (14 female, mean age 24.7, SD 3.1, range 21–33) approximately 8 months later (8.4 months, SD 1.2).

#### Memory interview

Participants were presented with the 16 photographs and cue phrases associated with the autobiographical memories in Experiment 1 and were asked to describe in as much detail as possible the specific event that they had recalled previously. General probes were given by the interviewer when appropriate (e.g., ‘what else can you remember about this event?’). The interviewer availed of summarised transcripts from Experiment 1 to verify the same memory and details were being recalled. Participants then rated each memory on the same characteristics assessed in Experiment 1. The memory interview during Experiment 2 was also recorded and transcribed.

#### Behavioural analyses

The analysis of subjective and objective ratings for Experiment 2 followed exactly the same procedure as Experiment 1. The extent to which subjective ratings for the same memory had changed between Experiment 1 and Experiment 2 was assessed using a two-way (experiment × time period) repeated-measures ANOVA with Bonferroni-corrected paired *t* tests. Differences in objective memory ratings across experiments were analysed using a two (experiment) × two (detail) × eight (time period) repeated-measures ANOVA with Bonferroni-corrected paired *t* tests.

#### Task during fMRI scanning

Participants returned approximately 1 week later for the fMRI scan (mean 5.5 days, SD 3.7). Prior to scanning, only 1 reminder training trial per memory was deemed necessary, given the prior experience of performing the task in Experiment 1. The scanning task remained unchanged from Experiment 1, aside from the rerandomisation of trials within each session. When the least vivid trials were excluded, the mean number of trials (/6) selected for analysis from each time period was as follows: 8 M: 5.94 (SD 0.25), 12 M: 5.97 (SD 0.13), 16 M: 5.88 (SD 0.29), 20 M: 5.88 (SD 0.29), 24 M: 5.94 (SD 0.25), 28 M: 5.94 (SD 0.17), 32 M: 5.84 (SD 0.40), 68 M: 5.81 (SD 0.36).

#### MRI data acquisition

Structural and functional data were acquired using the same scanner and scanning sequences as Experiment 1. However, the prior acquisition of the partial-volume structural MRI scans negated the need to include these in the protocol of Experiment 2.

#### MRI data preprocessing

fMRI data were preprocessed using the same pipeline as Experiment 1, with the additional step of coregistering the functional scans of Experiment 2 to the structural scans of Experiment 1, which enabled the use of the vmPFC masks from Experiment 1. First-level GLMs of each recall trial were constructed in an identical manner to Experiment 1.

#### RSA

RSA of the Experiment 2 fMRI data was conducted in an identical manner to Experiment 1. The average number of voxels analysed in the vmPFC across the two sets of memories for all participants was 5,228 (SD 1,765). To ascertain whether the observed neural representation scores had changed between Experiments 1 and 2, a two-way (experiment × time period) repeated-measures ANOVA was performed. To investigate if these changes mirrored the predictions generated by the original data, paired *t* tests were performed between the neural representation scores for each memory from Experiment 1 and Experiment 2, one-tailed if there was a hypothesised increase or decrease.

## Supporting information

S1 Table Behavioural data (mean, SD) for Experiment 1 (*n* = 30). Subjective ratings of memory characteristics and objective ratings of memory content for Experiment 1 (relates to Figs 1 and 2).
(DOCX)

S2 Table Neural representation scores (mean, SD) for other brain regions in Experiment 1 (*n* = 30). (DOCX)

S3 Table Behavioural data (mean, SD) for Experiment 2 (*n* = 16). Subjective ratings of memory characteristics and objective ratings of memory content for Experiment 2 (relates to Figs 6 and 7).
(DOCX)

S1 Fig RSM of within- and between-time-point pattern similarity values for Experiment 1. Each cell in this matrix contains the group mean pattern similarity score between memories from all sampled time points, averaged across the two memory sets. The values along the diagonal represent the within-memory similarity for each time point. Off-diagonal values indicate the correlation of neural patterns between memories of different ages, which are subsequently averaged to produce the baseline ‘between-memory’ value and subtracted from the ‘within-memory’ correlation to produce a neural representation score. For ease of visual inspection, all values are rank transformed, scaled between 0 and 1, and colour coded to indicate the magnitude of pattern similarity. RSM, representational similarity matrix.
(TIFF)

S2 Fig Box plot of neural representation scores for Experiment 1. Boxes represent 25th to 75th percentiles around the median; whiskers represent minimum and maximum values; means are indicated by solid circles (see S8 Data for individual participant numerical values).
(TIFF)

S3 Fig Within- versus between-time-point pattern similarity for Experiment 1. Time-dependent changes in neural representation scores were driven by within- rather than between-memory scores (see S9 Data for individual participant numerical values).
(TIFF)

S4 Fig Results of the group vmPFC searchlight analysis in MNI space for Experiment 1. (A) Colour-coded areas represent the FWE-corrected T-statistic in which within-memory detectability was higher than between-memory detectability across participants. (B) Comparison of memory detectability across time points within this functionally defined area, showing highly similar results to the whole ROI analysis in native space (see S10 Data for individual participant numerical values). FWE, family-wise error; MNI, Montreal Neurological Institute; ROI, region of interest; vmPFC, ventromedial prefrontal cortex.
(TIFF)

S1 Data Individual participant values underlying Fig 1B–1F. (XLSX)

S2 Data Individual participant values underlying Fig 2. (XLSX)

S3 Data Individual participant values underlying Fig 4. (XLSX)

S4 Data Individual participant values underlying Fig 5. (XLSX)

S5 Data Individual participant values underlying Fig 6B–6F. (XLSX)

S6 Data Individual participant values underlying Fig 7. (XLSX)

S7 Data Individual participant values underlying Fig 8. (XLSX)

S8 Data Individual participant values underlying S2 Fig. (XLSX)

S9 Data Individual participant values underlying S3 Fig. (XLSX)

S10 Data Individual participant values underlying S4B Fig. (XLSX)

S11 Data Individual participant additional subjective ratings from Experiment 1. (XLSX)

S12 Data Individual participant additional subjective ratings from Experiment 2. (XLSX)

S13 Data Individual participant values underlying S2 Table. (XLSX)

## Abbreviations

BA: Brodmann area
EPI: echo planar imaging
fMRI: functional magnetic resonance imaging
ICC: intraclass correlation
MNI: Montreal Neurological Institute
MVPA: multivoxel pattern analysis
PE: phase-encoding
ROI: region of interest
RSA: representational similarity analysis
RSM: representational similarity matrix
SNR: signal-to-noise ratio
TE: echo time
TR: volume repetition time
vmPFC: ventromedial prefrontal cortex
