# Supplementary material for: Nonmonotonic recruitment of ventromedial prefrontal cortex during remote memory recall
Source: PLoS Biol. 2018 Jul 2;16(7):e2005479. doi: 10.1371/journal.pbio.2005479 (PMC6044544; doi:10.1371/journal.pbio.2005479)
Supplement: S2 Table — (DOCX) [file pbio.2005479.s002.docx]

**Neural Representation** 0.5M 4M 8M 12M 16M 20M 24M 60M

Precuneus **.012 (.017)*** .011 (.020)** .015 (.013)*** .013 (.012)*** .011 (.015)*** .013 (.014)*** .016 (.018)*** .016 (.017)*****

Lateral temporal cortex **.004 (.011)* .007 (.013)* .005 (.010)** .007 (.012)**** .003 (.009) **.007 (.009)*** .007 (.013)** .007 (.011)*****

Parahippocampal cortex .004 (.011) .003 (.011) .005 (.013) **.005 (.013)*** .003 (.013) **.006 (.014)*** **.008 (.009)***** **.011 (.015)*****

Retrosplenial cortex .000 (.014) .001 (.018) .005 (.019) .005 (.015) **.006 (.015)*** .006 (.017) .004 (.017) **.008 (.017)***

Temporal pole .003 (.009) .002 (.012) .003 (.012) .003 (.009) .001 (.010) .002 (.009) **.006 (.009)** .008 (.008)*****

Entorhinal/Perirhinal cortex .002 (.012) .004 (.013) .000 (.012) .002 (.011) -.003 (.015) .000 (.014) .001 (.012) **.006 (.013)***

Hippocampus .001 (.007) .000 (.010) .002 (.011) .003 (.010) -.001 (.012) .001 (.010) .001 (.009) **.004 (.008)****

Asterisks indicate detectability from chance (* p < 0.05, ** p < 0.01, *** p < 0.001).
