# Supplementary material for: Nonmonotonic recruitment of ventromedial prefrontal cortex during remote memory recall
Source: PLoS Biol. 2018 Jul 2;16(7):e2005479. doi: 10.1371/journal.pbio.2005479 (PMC6044544; doi:10.1371/journal.pbio.2005479)
Supplement: S3 Table — Subjective ratings of memory characteristics and objective ratings of memory content for Experiment 2 (relates to Figs 6 and 7). (DOCX) [file pbio.2005479.s003.docx]

**A.**  **Subjective ratings** (0.5M) 8M (4M) 12M (8M) 16M (12M) 20M (16M) 24M (20M) 28M (24M) 32M (60M) 68M

Vividness 3.47 (0.72) 3.41 (0.84) 3.34 (0.68) 3.19 (0.79) 3.09 (0.66) 3.31 (0.77) 3.22 (0.71) 3.09 (0.80)

Detail 3.22 (0.63) 3.25 (0.84) 3.13 (0.56) 2.84 (0.65) 3.06 (0.66) 3.09 (0.76) 2.97 (0.62) 2.75 (0.80)

Difficulty 2.34 (0.60) 2.19 (0.85) 2.22 (0.82) 2.28 (0.58) 2.25 (0.63) 2.22 (0.75) 2.28 (0.97) 2.25 (0.61)

Personal significance 2.97 (0.69) 3.13 (0.74) 3.22 (0.66) 2.78 (0.75) 2.84 (0.87) 2.94 (0.87) 2.66 (0.79) 3.00 (0.86)

Valence 4.31 (0.54) 4.28 (0.63) 4.28 (0.60) 4.06 (0.75) 4.22 (0.68) 4.03 (0.74) 3.97 (0.56) 4.38 (0.65)

Active(1)/static event(2) 1.31 (0.31) 1.41 (0.42) 1.38 (0.34) 1.47 (0.43) 1.19 (0.31) 1.34 (0.47) 1.34 (0.40) 1.28 (0.31)

Self(1)/other perspective(2) 1.09 (0.20) 1.13 (0.29) 1.13 (0.29) 1.16 (0.30) 1.00 (0.00) 1.06 (0.17) 1.16 (0.24) 1.09 (0.27)

Recall frequency 2.03 (0.69) 2.00 (0.80) 1.78 (0.75) 1.72 (0.45) 1.84 (0.77) 1.81 (0.70) 1.50 (0.48) 1.53 (0.59)

Consistency 3.94 (0.36) 3.94 (0.44) 3.91 (0.61) 3.88 (0.67) 3.91 (0.55) 4.00 (0.58) 3.81 (0.63) 3.72 (0.71)

**B. Objective scores**

Internal details 18.75 (7.77) 17.56 (6.83) 17.59 (7.33) 18.66 (7.88) 19.63 (7.72) 20.31 (8.00) 17.06 (6.53) 16.03 (6.69)

External details 6.25 (4.27) 5.66 (4.06) 5.91 (2.78) 6.47 (4.46) 7.16 (3.34) 9.28 (5.40) 8.41 (5.18) 6.66 (4.36)
