# Supplementary figures and images for: Nonmonotonic recruitment of ventromedial prefrontal cortex during remote memory recall
Source: PLoS Biol. 2018 Jul 2;16(7):e2005479. doi: 10.1371/journal.pbio.2005479 (PMC6044544; doi:10.1371/journal.pbio.2005479)

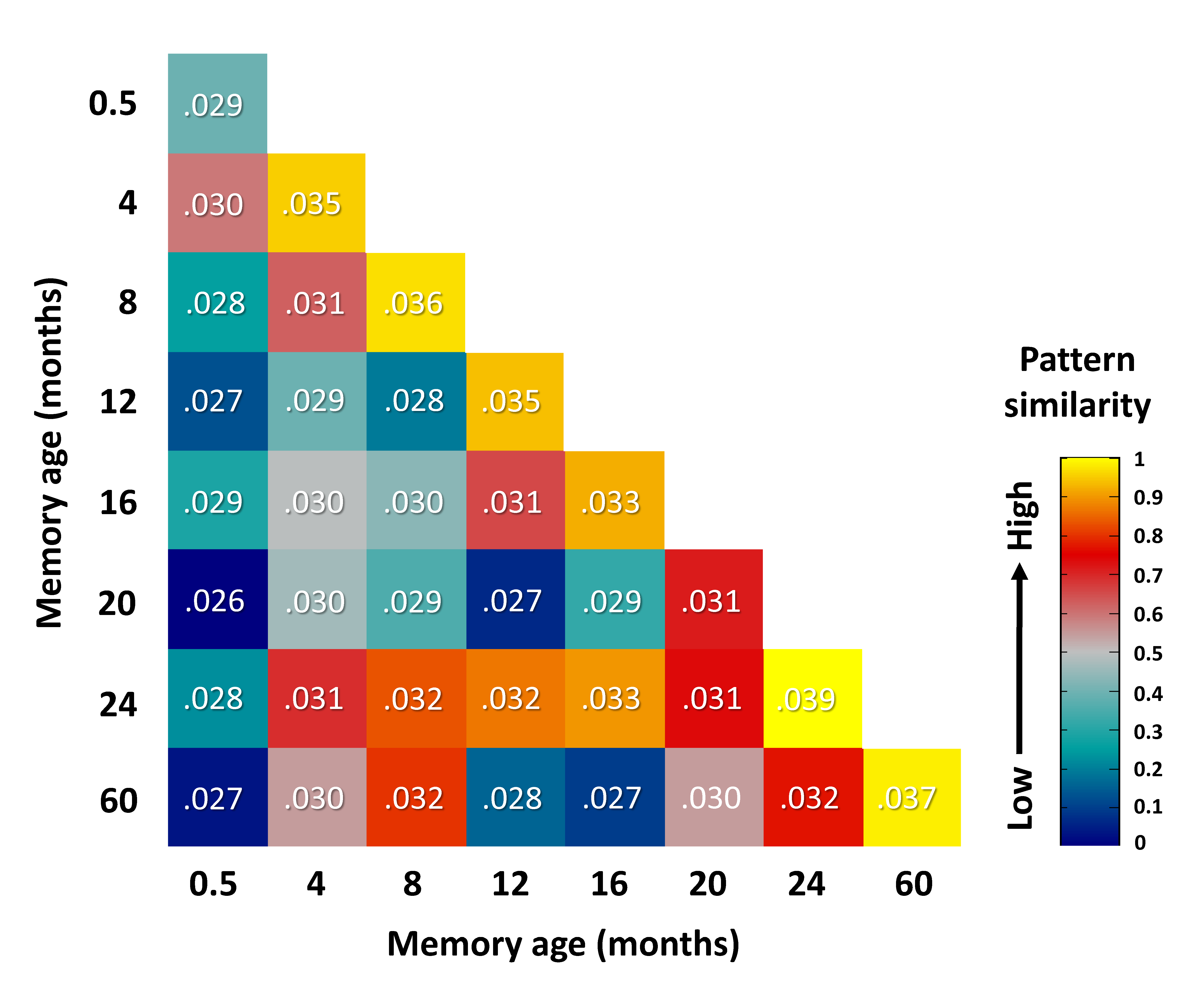

Supplement: S1 Fig — Each cell in this matrix contains the group mean pattern similarity score between memories from all sampled time points, averaged across the two memory sets. The values along the diagonal represent the within-memory similarity for each time point. Off-diagonal values indicate the correlation of neural patterns between memories of different ages, which are subsequently averaged to produce the baseline ‘between-memory’ value and subtracted from the ‘within-memory’ correlation to produce a neural representation score. For ease of visual inspection, all values are rank transformed, scaled between 0 and 1, and colour coded to indicate the magnitude of pattern similarity. RSM, representational similarity matrix. (TIFF) [file pbio.2005479.s004.tiff]

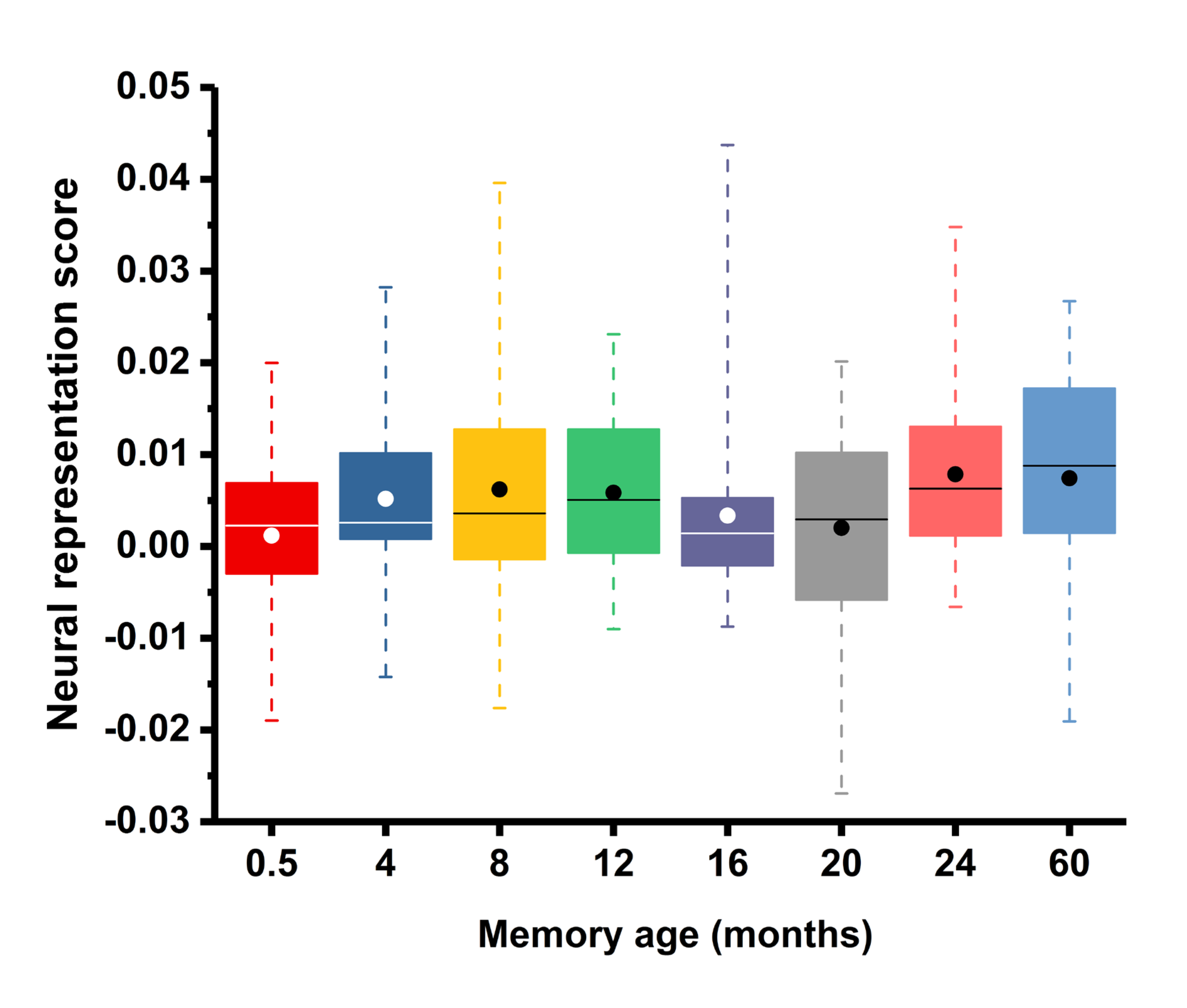

Supplement: S2 Fig — Boxes represent 25th to 75th percentiles around the median; whiskers represent minimum and maximum values; means are indicated by solid circles (see S8 Data for individual participant numerical values). (TIFF) [file pbio.2005479.s005.tiff]

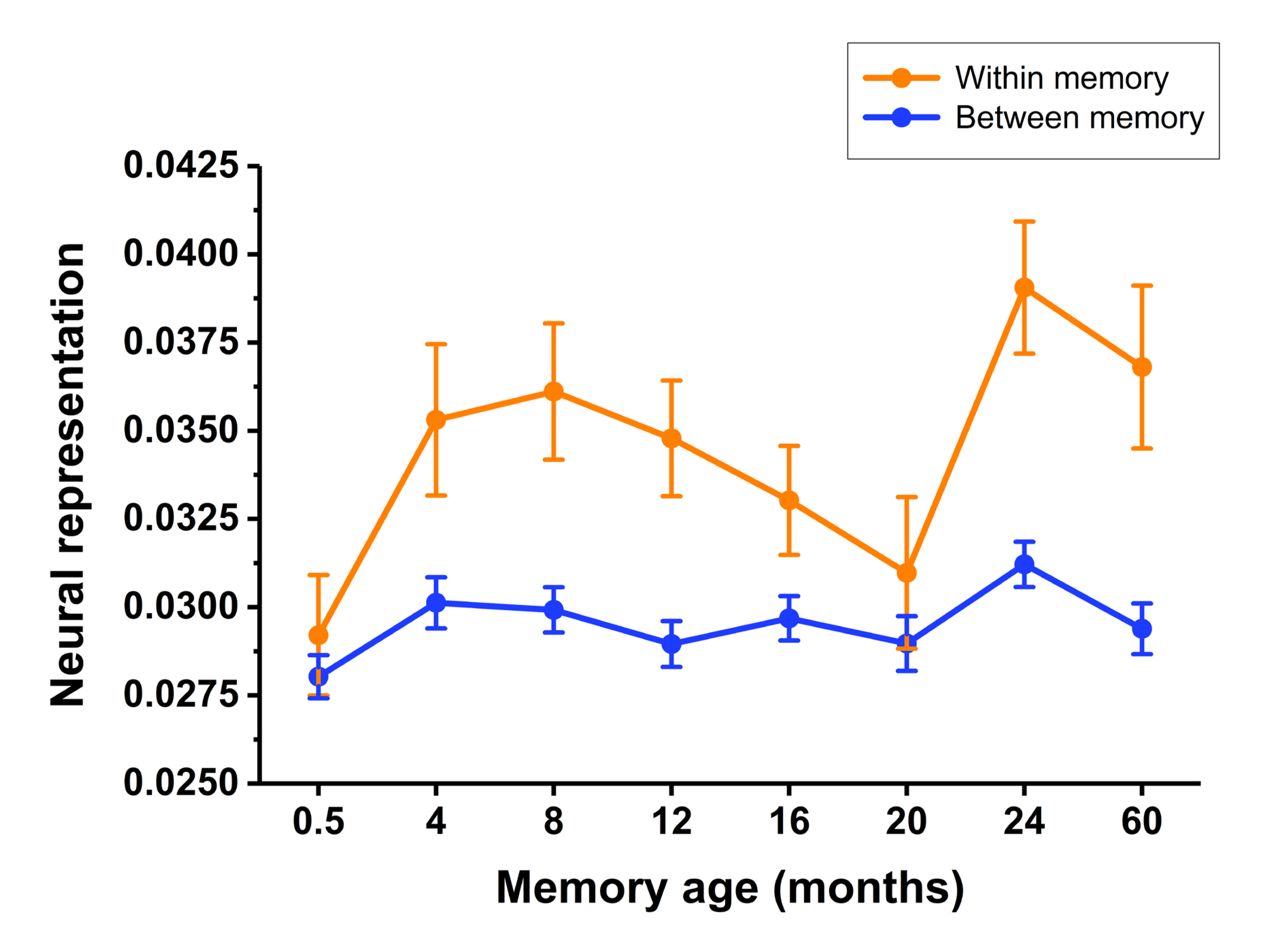

Supplement: S3 Fig — Time-dependent changes in neural representation scores were driven by within- rather than between-memory scores (see S9 Data for individual participant numerical values). (TIFF) [file pbio.2005479.s006.tiff]

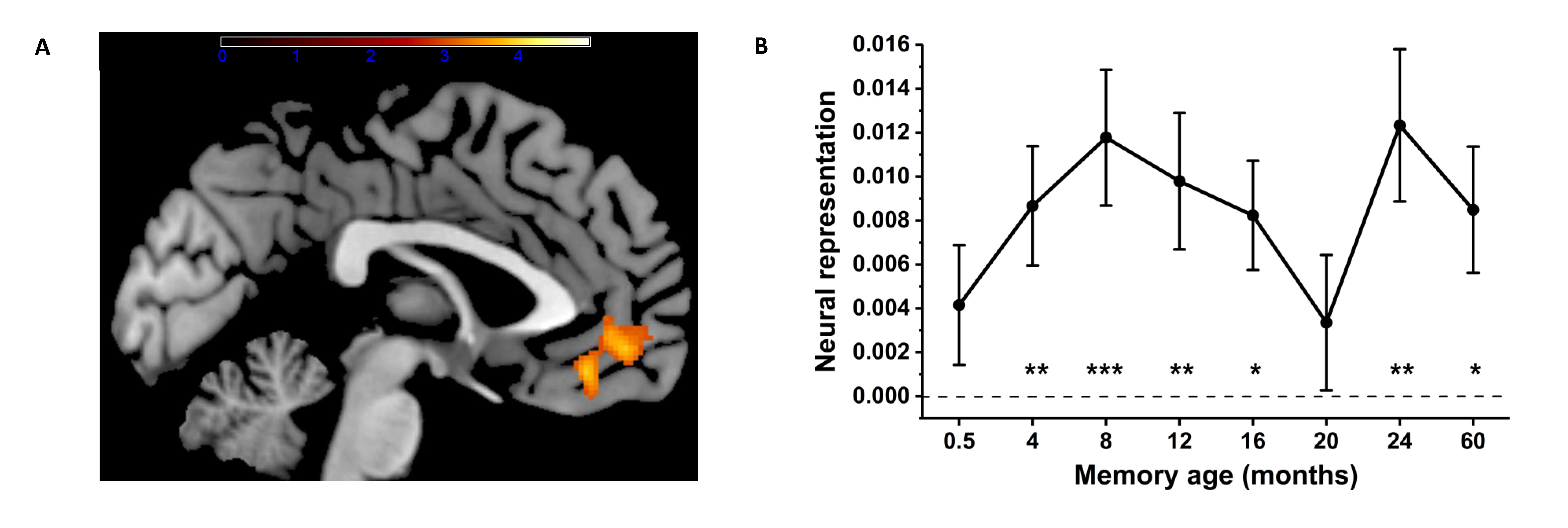

Supplement: S4 Fig — (A) Colour-coded areas represent the FWE-corrected T-statistic in which within-memory detectability was higher than between-memory detectability across participants. (B) Comparison of memory detectability across time points within this functionally defined area, showing highly similar results to the whole ROI analysis in native space (see S10 Data for individual participant numerical values). FWE, family-wise error; MNI, Montreal Neurological Institute; ROI, region of interest; vmPFC, ventromedial prefrontal cortex. (TIFF) [file pbio.2005479.s007.tiff]
